# Recurrent Modular Assembly Drives Independent Origins of Group II Introns Encoding LAGLIDADG Homing Endonucleases

**DOI:** 10.1093/gbe/evag114

**Published:** 2026-05-06

**Authors:** Feng Liu, Shuangle Jin, Qingbao Du

**Affiliations:** Laboratory of Marine Ecology and Environmental Sciences, Institute of Oceanology, Chinese Academy of Sciences (IOCAS), Qingdao 266071, China; Laboratory for Marine Ecology and Environmental Science, Qingdao Marine Science and Technology Center, Qingdao 266200, China; College of Marine Science, University of Chinese Academy of Sciences, Beijing 101408, China; Laboratory of Marine Ecology and Environmental Sciences, Institute of Oceanology, Chinese Academy of Sciences (IOCAS), Qingdao 266071, China; Laboratory for Marine Ecology and Environmental Science, Qingdao Marine Science and Technology Center, Qingdao 266200, China; College of Marine Science, University of Chinese Academy of Sciences, Beijing 101408, China; Tsingtao SINOVA-HK Biotechnology Co., Ltd., Qingdao 266400, China

**Keywords:** LAGLIDADG homing endonucleases, mitochondrial introns, RNA–protein evolution, modular evolution, mobile genetic elements

## Abstract

Group II introns are catalytic RNAs that typically mobilize with intron-encoded reverse transcriptase/maturase (RT/M) proteins, yet a small subset encodes LAGLIDADG homing endonucleases (LHEs). The evolutionary mechanisms underlying this atypical RNA–protein association remain poorly understood. Here, we analyze 753 LHE-encoding group II introns (group II-LHE introns) from green algae, fungi, and bacteria to investigate their origins and structural diversification. Our comparative analyses reveal that these introns are distributed across four deeply divergent RNA backbone subclasses (IIA1, IIB1, IIB2, and IIC) and form 13 lineage-specific intron families. Secondary-structure reconstruction reveals a conserved catalytic core together with extensive family-specific remodeling, including variable LHE insertion sites, recurrent loss of branch-point adenosine, and differential retention of exon-recognition elements. Phylogenetic incongruence between the RNA backbone and LHE phylogenies supports multiple independent acquisitions rather than descent from a single ancestral composite element. These results demonstrate that LHE modules have been repeatedly recruited onto distinct group II intron scaffolds, highlighting recurrent modular assembly as a key mechanism in the evolution of RNA–protein mobile elements. Our study provides a unified framework for understanding the origin, diversification, and modular evolution of LHE-encoding group II introns.

Significance Group II introns typically mobilize with intron-encoded reverse transcriptase/maturase (RT/M) proteins, yet a small subset carries LAGLIDADG homing endonucleases (LHEs). By analyzing 753 such elements across green algae, fungi, and bacteria, we show that LHE-encoding group II introns do not reflect a single linear evolutionary trajectory. Instead, they occur on multiple deeply divergent group II intron RNA backbones and show strong incongruence between RNA and LHE phylogenies, consistent with repeated modular recruitment of LHEs into distinct ribozyme scaffolds. These results highlight the exceptional plasticity of group II intron RNAs and support modular recombination as a key mechanism shaping the evolution of RNA–protein mobile elements.

## Introduction

Group II introns are large catalytic RNAs that act as self-splicing ribozymes and, in many cases, mobile genetic elements (Belfort 2015; Manigrasso et al. 2020). They are widely distributed in bacteria, archaea, and eukaryotic organellar genomes (Lambowitz and Zimmerly 2004; Mukhopadhyay and Hausner 2021) as well as phage genomes (Merk et al. 2025), and are thought to be evolutionary ancestors of spliceosomal introns and non-LTR retrotransposons (Piccirilli and Staley 2019; Monachello et al. 2021). Despite extensive sequence divergence, group II introns retain a conserved six-domain RNA secondary structure (DI–DVI) (Michel and Ferat 1995; Liu et al. 2025). Based on RNA structural features and exon-recognition mechanisms, group II introns are classified into several subclasses, including IIA (A1 and A2), IIB (B1 and B2), IIC, IIE, and IIF (Toor et al. 2001; Pyle 2016).

Canonical group II introns typically encode a multifunctional intron-encoded protein (IEP), consisting of reverse transcriptase (RT) and maturase (M) domains, and often a DNA endonuclease domain, which facilitates RNA splicing and promotes intron mobility through target-primed reverse transcription (Lambowitz and Zimmerly 2011; Costa 2022). However, a small subset of mitochondrial group II introns lacks a canonical RT/M-type IEP and instead encodes LAGLIDADG homing endonucleases (LHEs) (Toor and Zimmerly 2002; Mullineux et al. 2010, 2011; Sethuraman et al. 2013; Liu et al. 2026). These atypical elements are hereafter referred to as LHE-encoding group II introns or group II-LHE introns and represent a deviation from the canonical architecture of group II introns (Toor and Zimmerly 2002; Zimmerly and Semper 2015). LHEs represent the most abundant and best-characterized class of homing endonucleases in eukaryotic organellar genomes and are most commonly encoded by group I introns (Chevalier and Stoddard 2001; Stoddard 2014). They are highly site-specific DNA endonucleases that typically contain one or two conserved LAGLIDADG motifs and function either as homodimers or monomers (Yahara et al. 2009; Stoddard 2011). In contrast, LHEs embedded within group II introns are uncommon, and the evolutionary origin and mechanistic basis of this RNA–protein association remain poorly understood.

**Fig. 1.**
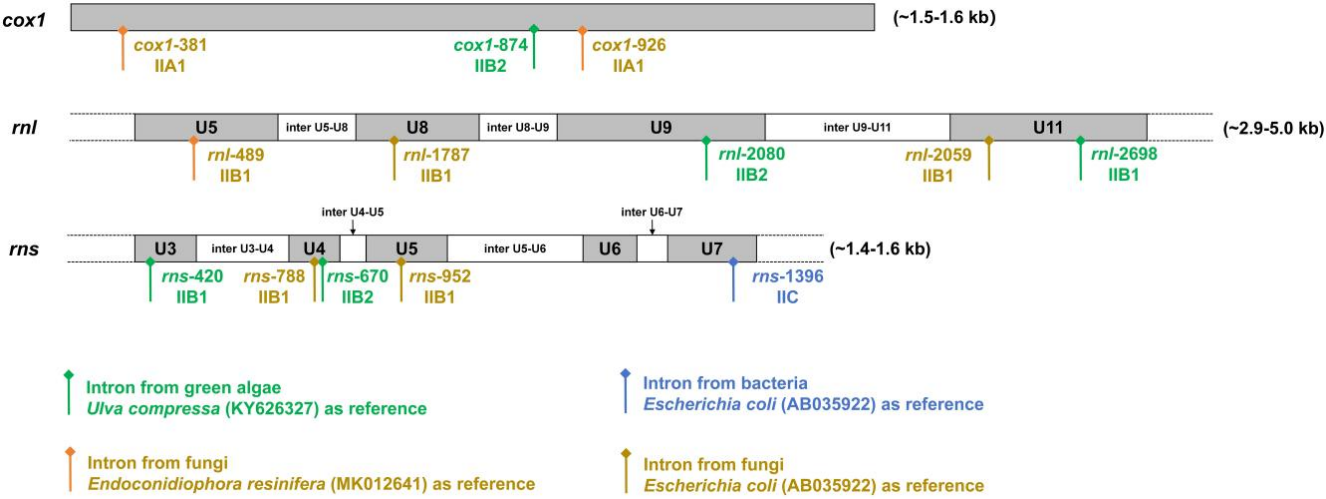
Insertion sites of the 13 identified group II-LHE intron families detected in three genes (*cox1*, *rnl*, and *rns*). These boxes represent the full-length *cox1* gene and partial rRNA genes, with universally conserved sequences U5–U11 within *rnl* and U3–U7 within *rns*. Numbers following gene names indicate intron insertion sites relative to the reference gene sequence, and intron subclasses (IIA1, IIB1, IIB2, and IIC) are indicated below each insertion site. Colored markers denote homologous introns identified from different reference sources: green, green algae (*Ulva compressa*, KY626327); orange, fungi (*Endoconidiophora resinifera*, MK012641); blue, bacteria (*Escherichia coli*, AB035922); and yellow, fungal introns represented by reference sequences reported in *E. coli* (AB035922). The approximate lengths of genes (excluding introns) observed in the analyzed genomes are indicated on the right.

To date, group II-LHE introns have been reported primarily from mitochondrial genomes of fungi and green algae (Toor and Zimmerly 2002; Pfeifer et al. 2012; Liu et al. 2020), as well as from a limited number of bacterial lineages (Salman et al. 2012). In fungi, these introns have been predominantly identified in certain phylogenetic lineages within the phyla Ascomycota, Basidiomycota, and Mucoromycota, and are largely confined to a small number of highly conserved mitochondrial housekeeping genes, such as the large and small subunit ribosomal RNA genes (*rnl* and *rns*) (Toor and Zimmerly 2002; Megarioti and Kouvelis 2020). Some members are inserted at specific sites (e.g. *rnl*-1787, *rnl*-2059, *rns*-788, and *rns*-952) and have been examined in detail (Fig. 1) (Mullineux et al. 2010, 2011; Sethuraman et al. 2013; Hafez et al. 2014). In addition, a group IIC-LHE intron has been described in the 16S rRNA (*rns*) gene of large sulfur bacteria, *Thiomargarita namibiensis* (Salman et al. 2012). In green algae, previous studies have reported five mitochondrial group II-LHE intron families (*cox1*-874, *rnl*-2080, *rnl*-2698, *rns*-420, and *rns*-670) in *Ulva* species (Ulvophyceae, Chlorophyta) (Fig. 1) (Liu et al. 2020, 2022a, 2026). In addition, another group II-LHE intron was detected at position 1911 within the mitochondrial *rnl* gene of *Gloeotilopsis planctonica* (Turmel et al. 2016). No group II-LHE introns have been reported from mitochondrial genomes of other eukaryotes or from chloroplast genomes of photosynthetic eukaryotes to date.

Previous studies on fungal group II-LHE introns proposed that these elements originated from IIB1-type group II introns through invasion of LHE genes into the intron RNA scaffold (Toor and Zimmerly 2002; Sethuraman et al. 2013). However, LHE integration sites vary among intron families, most often within the “safe haven” region of the intron RNA scaffold, classically domain IV (DIV), which commonly accommodates intronic open reading frames (ORFs) but with documented exceptions (Mullineux et al. 2010, 2011). Moreover, while most group II-LHE introns encode LHEs with two LAGLIDADG motifs (Li et al. 2011; Hafez et al. 2014), single-motif variants and degenerated or truncated ORFs have also been observed (Sethuraman et al. 2013), consistent with repeated gain/loss and degradation dynamics typical of mobile intron-associated ORFs (Pyle 2016). Although previous studies have proposed that fungal group II-LHE introns originated from IIB1-type ancestors (Toor and Zimmerly 2002; Sethuraman et al. 2013) and the LHE-encoding intron families in *Ulva* species originated from at least two independent invasion events within IIB1 and IIB2 lineages (Liu et al. 2026), these inferences were based on limited, lineage-specific sampling and have not been tested within a broader phylogenetic framework.

These observations suggest repeated invasion of distinct LHE modules followed by lineage-specific remodeling. However, it remains unclear whether group II-LHE introns originated from a single ancestral composite element that subsequently diversified, or instead arose repeatedly through independent recruitment of LHEs into distinct group II intron RNA scaffolds. Here, we perform a backbone-level, cross-kingdom evolutionary analysis of group II-LHE introns across green algae, fungi, and bacteria. By integrating comparative RNA secondary-structure reconstruction, phylogenetic analyses of conserved intron RNA cores, LHE protein evolution, and topology tests of alternative hypotheses, we evaluate competing models of single versus multiple origins.

## Results

### Taxonomic Distribution of Group II-LHE Introns

**Fig. 2.**
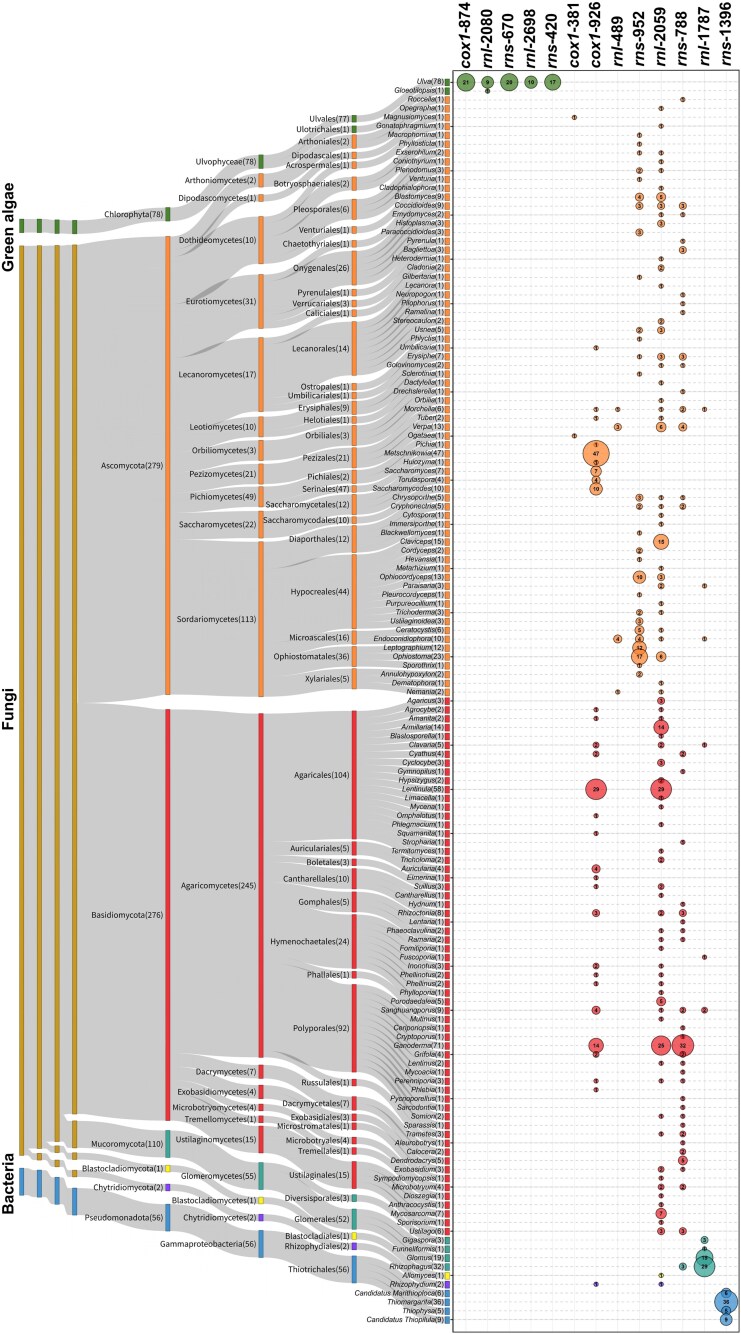
Taxonomic and quantitative distributions of group II-LHE introns. Sankey diagram summarizing the phylogenetic distribution of identified group II-LHE intron families across major lineages of green algae, fungi, and bacteria, resolved hierarchically from phylum to genus. The width of each flow is proportional to the number of genera that contain group II-LHE introns assigned to the corresponding taxonomic rank. Numbers in parentheses indicate group II-LHE intron counts in each node. Bubble plot showing the distribution of each group II-LHE family among genera. Bubble area is proportional to the number of group II-LHE introns detected for each genus and each intron family. Numbers inside bubbles denote intron counts for lineage categories. Bubble colors match the lineage categories in the Sankey diagram, helping to visually link each genus with its taxonomic lineage.

A total of 753 LHE-encoding group II introns were identified and classified into 13 distinct intron families based on insertion site homology (Fig. 1) and sequence similarity. These introns exhibit a highly uneven phylogenetic distribution and occur almost exclusively in mitochondrial genomes of green algae and fungi, with only a limited presence in bacterial genomes (Fig. 2). This pattern persists despite the extensive availability of mitochondrial and bacterial rRNA gene sequences in current genomic databases, suggesting that it cannot be solely explained by incomplete sampling, although database bias cannot be excluded.

In green algae, five mitochondrial intron families (*cox1*-874, *rnl*-2080, *rnl*-2698, *rns*-420, and *rns*-670) were detected within the Ulvales/Ulotrichales lineage of Ulvophyceae. Four of these families were restricted to the genus *Ulva*, whereas *rnl*-2080 was additionally detected in *Gloeotilopsis* (Fig. 2). The intron reported previously at position 1911 in the *rnl* gene of *G. planctonica* belongs to this family (Turmel et al. 2016).

In fungi, mitochondrial group II-LHE introns were distributed across five fungal phyla (Ascomycota, Basidiomycota, Mucoromycota, Chytridiomycota, and Blastocladiomycota). Alongside four previously recognized families (*rnl*-1787, *rnl*-2059, *rns*-788, and *rns*-952), three new families (*cox1*-381, *cox1*-926, and *rnl*-489) have been identified for the first time. Among these, *rnl*-2059 and *rns*-788 had the broadest distributions, occurring mainly in Ascomycota and Basidiomycota. In addition, *rnl*-2059 was detected in one genus within Blastocladiomycota and one within Chytridiomycota, while *rns*-788 was found in one genus within Mucoromycota (Fig. 2). In contrast, *cox1*-926 was observed in nine genera within Ascomycota, 19 genera within Basidiomycota, and one genus within Chytridiomycota, while *cox1*-381 was limited to two genera within Ascomycota. Notably, two families (*rns*-952 and *rnl*-489) were confined to Ascomycota, whereas *rnl*-1787 was enriched in Mucoromycota but occurred sporadically in Ascomycota and Basidiomycota.

In bacteria, only a single family (*rns*-1396) was detected. The insertion site of this bacterial intron differed from those observed in eukaryotic mitochondrial *rns* genes. The representatives occurred exclusively within four genera within the Thiotrichaceae lineage of Gammaproteobacteria (Fig. 2).

These data indicate that group II-LHE introns are rare, highly lineage-restricted mobile elements, suggesting that their current distribution may reflect multiple lineage-specific acquisition events rather than widespread vertical inheritance. This pattern persists even in lineages with extensive sampling of eukaryotic mitochondrial genomes and bacterial *rns* genes in currently available datasets.

### Conserved Catalytic Architecture of Group II-LHE Introns and Diversity of LHE Insertion

**Fig. 3.**
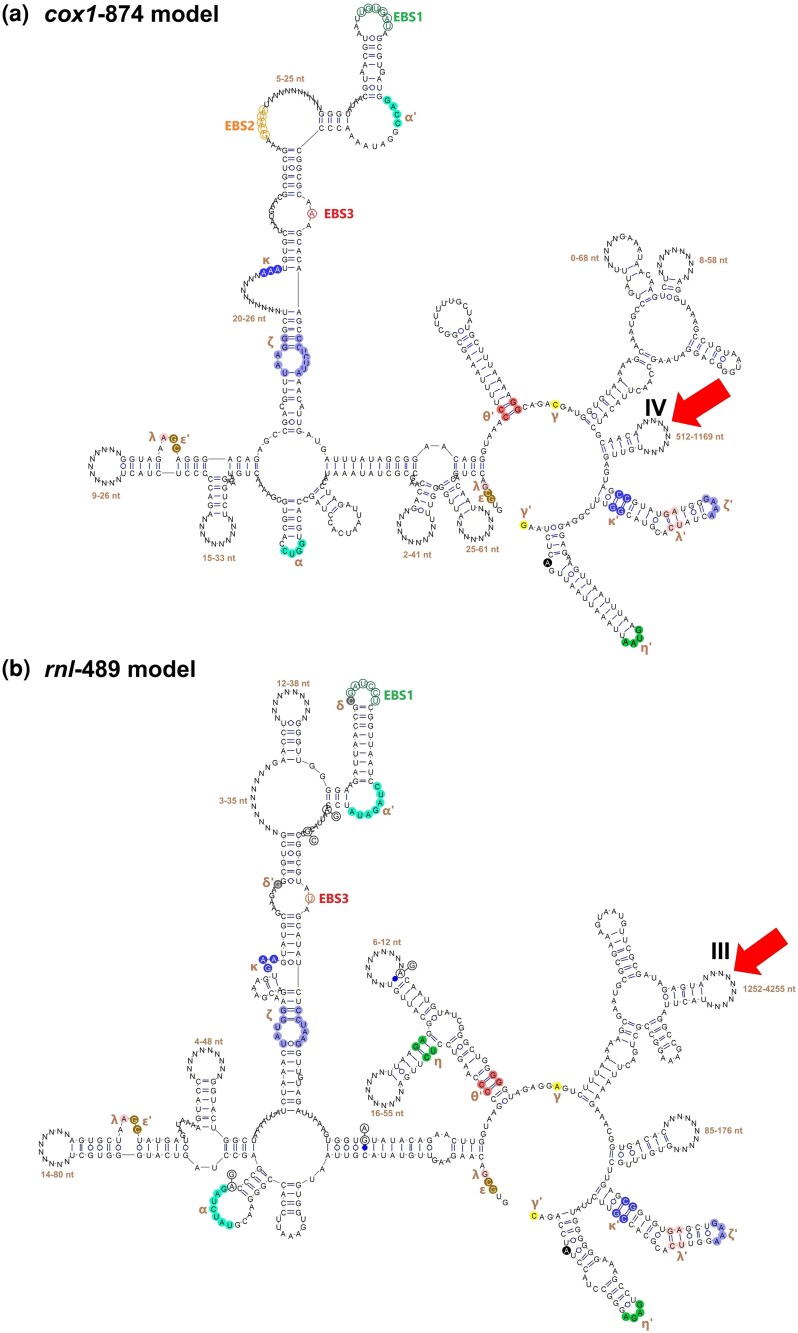

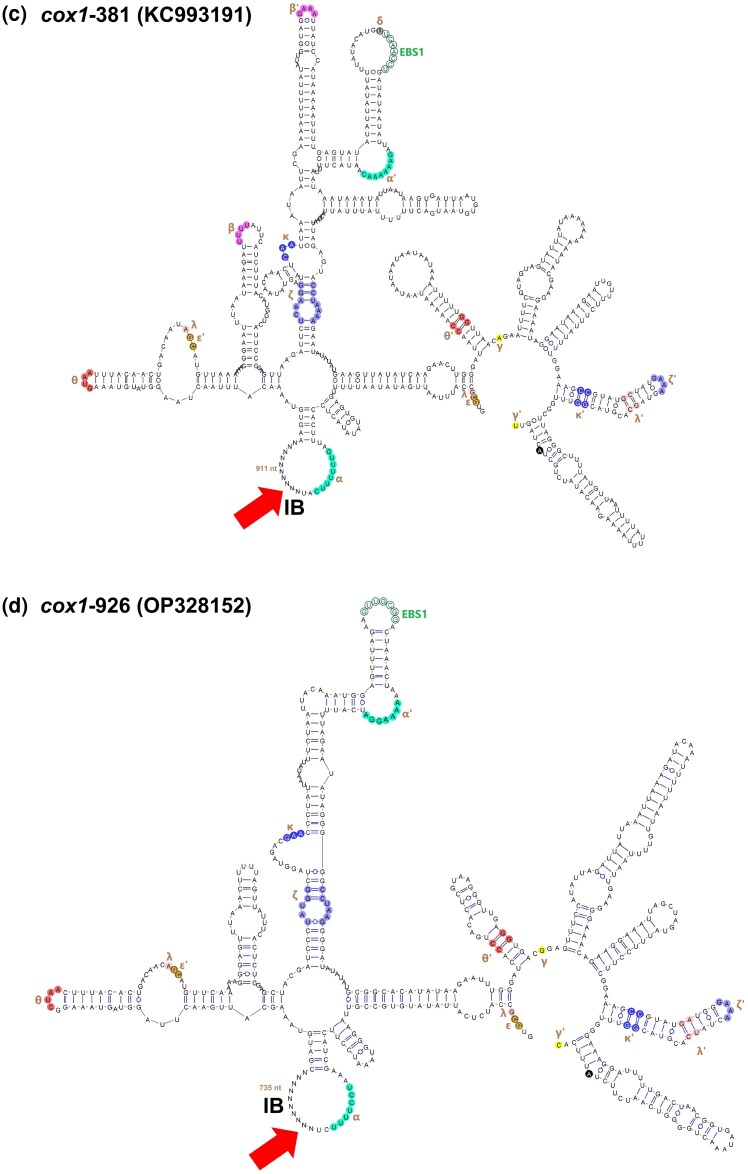
Predicted secondary structures of representative group II-LHE introns. Secondary-structure models are shown for group II-LHE introns, predicted using RNA structure prediction tools (RNAstructure or mfold). Representative intron families are shown: a) *cox1*-874; b) *rnl*-489; c) *cox1*-381 (GenBank KC993191); and d) *cox1*-926 (GenBank OP328152). Canonical group II intron domains (DI–DVI) are indicated on the structures. The exon-binding sites (EBS1–EBS3) are highlighted, which play a crucial role in the recognition of exon regions for efficient splicing. Red arrows mark the position of the LHE ORF insertion within the intron RNA scaffold, occurring in different structural regions such as DIV, DIII, or DIB, each with unique functional characteristics impacting exon recognition and intron splicing efficiency. Inserted sequences, including LHE ORFs, are indicated within variable regions of the introns. Distinct colors denote conserved regions and functionally important motifs across different intron types.

Despite extensive primary-sequence divergence, all 13 intron families retain the canonical six-domain secondary structure (DI–DVI) characteristic of group II introns (Fig. 3 and Figs. S1 to S3). Among these domains, DV represents the most conserved structural element and preserves the characteristic catalytic stem-loop configuration required for intron splicing. DV is 34 nucleotides long in most families, with the exception of *cox1*-874, which contains a slightly shorter DV of 32 nucleotides. The preservation of this catalytic domain across all families indicates that the conserved catalytic core of the ribozyme has been maintained despite extensive evolutionary diversification.

Outside the catalytic core, however, these introns display extensive structural heterogeneity, including domain expansions or reductions, heterogeneous LHE insertion sites, degeneration of LHE-coding regions, lineage-specific sequence accretions, and differential retention of predicted tertiary interaction motifs. These results demonstrate that group II-LHE introns share a conserved catalytic scaffold but exhibit extensive structural remodeling in peripheral domains, suggesting that ribozyme cores remain stable while mobility-related features evolve more rapidly.

The positions of LHE ORFs vary substantially among intron families (Fig. 3 and Figs. S1 to S3). The most frequent insertion site occurs in DIV, which accommodates LHE ORFs in all green algal families, three fungal families, and the bacterial *rns*-1396 family (Fig. 3a). However, several fungal families display alternative insertion contexts. In *rnl*-489 and *rns*-952, the LHE ORF occurs within domain III (DIII) (Fig. 3b), whereas in *cox1*-381 and *cox1*-926 it is located within the loop region of domain IB (DIB) (Fig. 3c and d). The distribution of LHE insertion sites across multiple structural domains indicates that LHE genes have been integrated repeatedly into different structural contexts of the group II intron scaffold.

### Classification based on RNA Secondary Structure and Lineage-Specific Structural Diversification

**Fig. 4.**
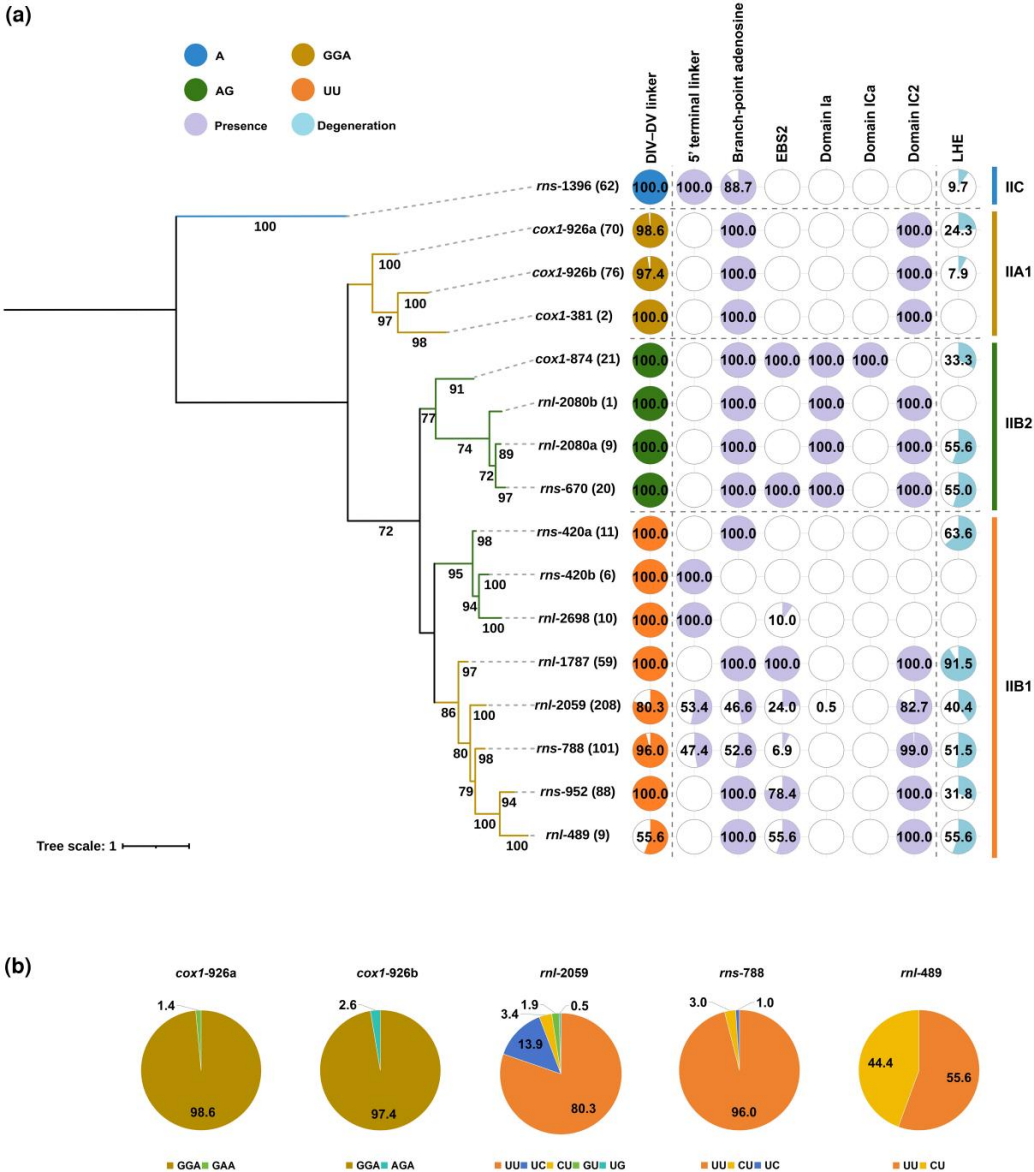
RNA-based phylogeny of group II-LHE intron families. a) ML phylogeny inferred from aligned group II-LHE intron RNA sequences, reconstructed using the best-fitting substitution model. The number of group II-LHE introns in each family is given in parentheses. Node support values (bootstrap percentages) are shown at internal branches. Branch lengths are drawn to scale (scale bar shown). The colored bars at the right indicate group II-LHE intron subclasses (IIA1, IIB1, IIB2, and IIC) based on RNA structural features and evolutionary clustering, consistent with the established RNA secondary-structure–based classification of group II-LHE introns. To the right of the tree, a presence/degeneration matrix summarizes key RNA structural features for each intron family. Circles indicate the proportion (%) of introns within each family exhibiting each feature, including the motifs at the DV-DVI linker (e.g. GGA in IIA1, UU in IIB1, AG in IIB2, and A in IIC), presence of 5′ terminal linker, the branch-point adenosine, EBS2, domains Ia, ICa, and IC2, and the proportion of degeneration in LHE ORFs (e.g. premature stop codons, frameshifts, or loss of catalytic residues). According to the topology of the evolutionary tree, differentiation occurred within some intron families, such as *cox1*-926a (found in Ascomycota) versus *cox1*-926b (found in Ascomycota, Basidiomycota, and Chytridiomycota), *rns*-420a (without 5′ terminal linker) versus *rns*-420b (with 5′ terminal linker), and *rnl*-2080a (found in *Ulva* species) versus *rnl*-2080b (found in *Gloeotilopsis* species). b) Proportions (%) of the DV-DVI linker motif variants for four intron families (*cox1*-926, *rnl*-2059, *rns*-788, and *rnl*-489), shown as pie charts. Slice sizes correspond to the frequency of each motif variant within each family, representing the diversity of DV-DVI linker motifs in each family.

Diagnostic structural characters allowed assignment of introns to established group II subclasses (IIA1, IIB1, IIB2, and IIC). Fungal *cox1*-381 and *cox1*-926 were classified as IIA1 based on a characteristic DII-DIII linker motif (CRGA), a distinct DIV-DV linker motif (GGA or its variants), and the absence of insertions in the 3′ strand of domain I(i)/I(ii). Ten families belonged to IIB subclasses and were further subdivided into IIB1 and IIB2 according to hallmark features, including distinct DIV-DV linker motifs (UU or its variants in IIB1 versus AG in IIB2) and the absence (IIB1) or presence (IIB2) of domain Ia in the 5′ strand of domain I(i)/I(ii). The bacterial *rns*-1396 family retained characteristic IIC features, including absence of domain IC2, diagnostic domain ID structure, the DII-DIII linker, and a conserved DIV-DV linker motif (A) (Fig. 4).

Comparative structural analyses further revealed multiple noncanonical features (Fig. 4). One notable example is the 5′ terminal linker, inserted between the 5′ exon and the conserved 5′ intron motif. This linker occurs in all members of several families (*rns*-1396, *rnl*-2698, and *rns*-420b) but appears only in a subset of others (*rnl*-2059 and *rns*-788). In many IIB1 families, the presence of this linker is associated with a noncanonical DVI lacking the typical branch-point adenosine, whereas families lacking the linker generally retain a canonical branch-point site.

Additional lineage-specific modifications include the gain or loss of structural domains within DI (Fig. 4). For example, the canonical domain Ia is retained primarily in IIB2 introns (*cox1*-874, *rnl*-2080, and *rns*-670), whereas domain IC2 is absent in several families, including *cox1*-874, *rnl*-2698, *rns*-420, and *rns*-1396. These structural features collectively indicate that group II-LHE introns have undergone extensive lineage-specific remodeling while preserving the conserved catalytic framework of the ribozyme core.

### Backbone-Level RNA Phylogeny of Group II-LHE Introns

Phylogenetic analyses of conserved intron RNA regions recovered four deeply divergent clades corresponding to established group II intron subclasses: IIA1, IIB1, IIB2, and IIC (Fig. 4 and Fig. S4). Rather than forming a single monophyletic group, group II-LHE introns are distributed across these four RNA backbone subclasses. The bacterial *rns*-1396 family forms a distinct IIC clade. Fungal *cox1*-381 and *cox1*-926 cluster within the IIA1 backbone. Five green algal families fall within the IIB subclasses: three families (*cox1*-874, *rnl*-2080, *rns*-670) group within IIB2, whereas two families (*rnl*-2698 and *rns*-420) cluster with five fungal families in the IIB1 clade. IIB1 and IIB2 were recovered as sister clades in the RNA-based phylogeny. These results reveal that LHE acquisition occurred on multiple deeply divergent group II intron scaffolds.

These phylogenetic patterns are congruent with classifications based on RNA secondary structure, demonstrating that group II-LHE introns occupy multiple deeply divergent evolutionary lineages rather than forming a single evolutionary radiation. To evaluate whether these introns could derive from a single ancestral composite element, we compared the best unconstrained phylogeny with constrained trees enforcing monophyly of all group II-LHE introns. Both AU and SH topology tests significantly rejected the monophyly-constrained hypothesis (*P* < 0.05), supporting the inference that these elements arose independently on multiple group II intron backbones.

### Evolution of EBS–IBS Recognition Interfaces and Insertion Sites

**Fig. 5.**
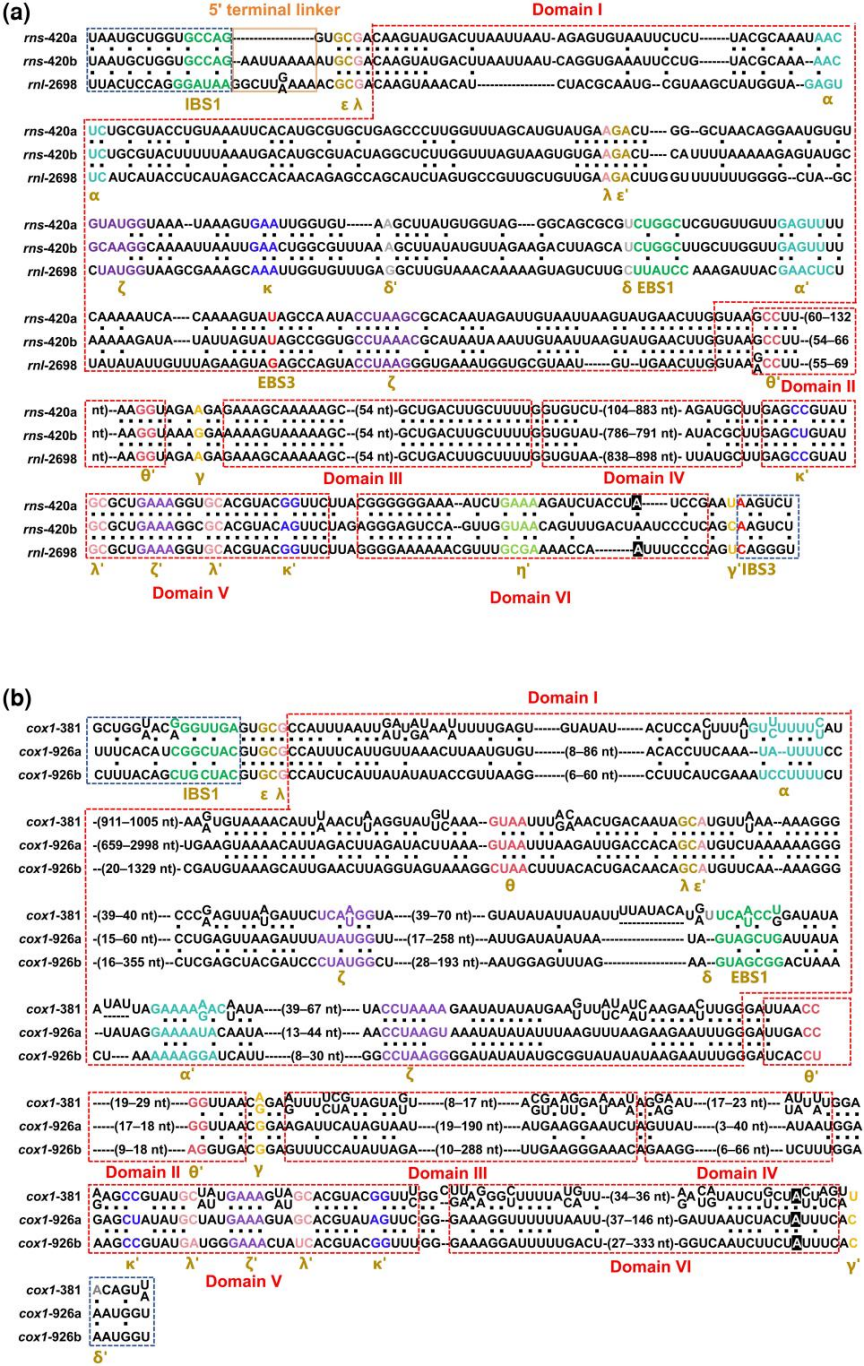
Secondary-structure–guided sequence alignment of mitochondrial group II-LHE introns from green algae and fungi. a) Group IIB1-LHE introns from green algae: *rns*-420a (without 5′ terminal linker), *rns*-420b (with 5′ terminal linker), and *rnl*-2698. b) Group IIA1-LHE introns from fungi: *cox1*-381, *cox1*-926a (found in Ascomycota), and *cox1*-926b (found in Ascomycota, Basidiomycota, and Chytridiomycota). Exon sequences at the 5′ and 3′ termini are shown in uppercase letters. Intron sequences were aligned manually according to conserved secondary structure domains. Canonical structural domains of group II introns (DI–DVI) are indicated with Roman numerals. Conserved tertiary interaction motifs, including EBS/IBS pairs (EBS1/IBS1, EBS2/IBS2, and EBS3/IBS3) and additional paired motifs (α–α′, β–β′, γ–γ′, κ–κ′, λ–λ′, δ–δ′, and ε–ε′), are labeled if present. The 5′ terminal linker is also indicated. Dashes represent alignment gaps introduced to maximize positional homology. Numbers in parentheses indicate nucleotide (nt) length variation among introns. Conserved nucleotides are color-coded according to their functional motif.

Comparative structural analyses revealed that intron families clustering closely in the phylogenetic tree share a conserved structural framework but also exhibit lineage-specific variation in EBS–IBS recognition interfaces. Two green algal IIB1-LHE intron families (*rns*-420 and *rnl*-2698) display highly similar structural organization across all six canonical domains (DI–DVI). Structural differences were mainly observed at the intron–exon boundary and in EBS motifs (Fig. 5a). Specifically, *rns*-420a lacked a 5′ terminal linker, whereas *rns*-420b and *rnl*-2698 possessed an additional 5′ terminal linker. In addition, the EBS2 motif was detected only in some members of the *rnl*-2698 family.

Two fungal mitochondrial IIA1-LHE intron families, *cox1*-381 and *cox1*-926, exhibited substantial sequence length variation, particularly within DI and DIV (Fig. 5b), reflecting insertions or deletions in peripheral regions of the intron structure. In contrast, their core structural motifs and tertiary interaction elements (ε–ε′, α–α′, γ–γ′, κ–κ′, λ–λ′, and δ–δ′) remained highly conserved. Similar structural patterns were observed in two IIB2-LHE intron families, *rnl*-2080 and *rns*-670 (Fig. S5a). However, two fungal IIB1-LHE intron families, *rns*-952 and *rnl*-489, exhibited pronounced sequence length variation within DIII (Fig. S5b), which harbors the LHE ORF.

In particular, loss of the EBS2 motif is widespread among group II-LHE introns (Tables S1 to S4). Only three families (*cox1*-874, *rns*-670, and *rnl*-1787) retained the complete set of EBS1–EBS3 motifs. In contrast, five families (*cox1*-381, *cox1*-926, *rnl*-2080, *rns*-420, and *rns*-1396) lack EBS2 entirely, despite belonging to four distinct intron subclasses. Intermediate retention patterns occur in several other families. For example, EBS2 is retained in more than half of the members in the *rnl*-489 and *rns*-952 families but in fewer than one quarter of the members of *rnl*-2059, *rnl*-2698, and *rns*-788.

**Fig. 6.**
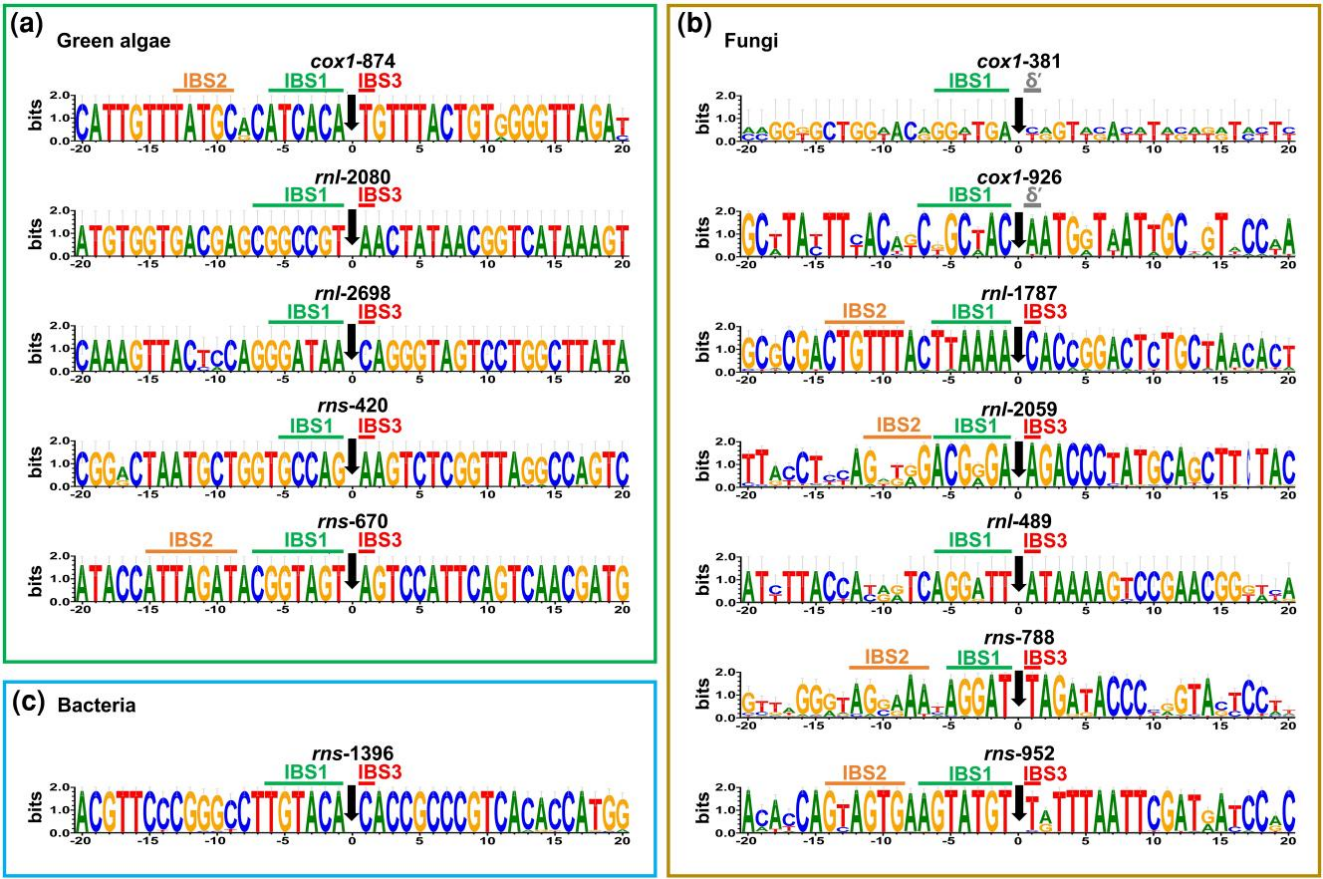
Sequence logos of insertion sites for group II-LHE intron families across green algae, fungi, and bacteria. Sequence logos showing nucleotide composition surrounding intron insertion sites in representative genes (*cox1*, *rnl*, and *rns*) across major lineages. The insertion position is indicated by a black arrow. Conserved intron-binding sites (IBS1, IBS2, and IBS3) and δ′ are highlighted with green, orange, red, and grey bars, respectively. The *x* axis indicates nucleotide positions relative to the insertion site (position 0), and the *y* axis shows information content (bits). a) Green algae. Sequence logos of insertion sites for five mitochondrial intron families from green algae, including *cox1*-874, *rnl*-2080, *rnl*-2698, *rns*-420, and *rns*-670. Conserved IBS1, IBS2, and IBS3 motifs are shown when present. b) Fungi. Sequence logos of insertion sites for seven mitochondrial intron families in fungi, including *cox1*-381, *cox1*-926, *rnl*-1787, *rnl*-2059, *rnl*-489, *rns*-788, and *rns*-952. Conserved IBS1, IBS2, IBS3, and δ′ motifs are shown when present. c) Bacteria. Sequence logo of the insertion site for the bacterial *rns*-1396 family, showing conserved IBS1 and IBS3 motifs.

Consistent with these differences in exon-recognition elements, insertion-site sequences also differ substantially among closely related intron families (Fig. 6). These observations suggest that rapid evolution of the EBS–IBS recognition interface may facilitate shifts in target specificity and promote the proliferation of introns across new genomic sites.

### LHE Phylogeny Reveals Modular Evolutionary Histories

A total of 410 intact LHE ORFs containing two LAGLIDADG motifs were detected in 11 families, and 69 complete LHE ORFs containing a single LAGLIDADG motif were found in two families (fungal *rnl*-1787 and bacterial *rns*-1396). In addition, 274 intron instances from 11 families contained degenerated LHE regions from which full-length, intact LHE sequences could not be recovered. Only two families, *cox1*-381 and *rnl*-2698, contained intact LHE ORFs in all members (Fig. 4).

**Fig. 7.**
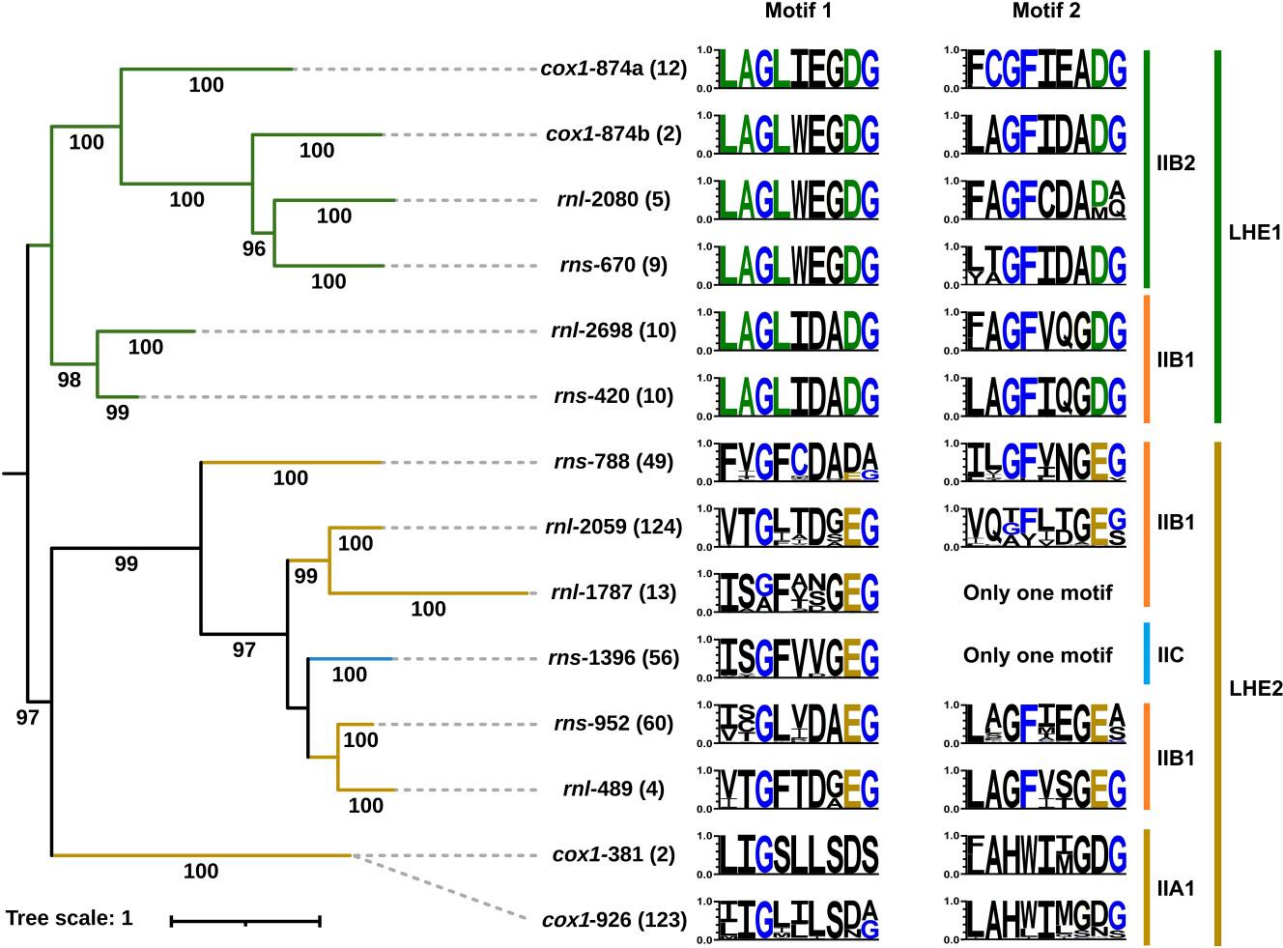
Phylogeny of LHEs encoded by group II-LHE introns and their conserved motif profiles. ML phylogeny of LHE amino acid sequences encoded by group II-LHE intron families, reconstructed using the best-fitting substitution model. Numbers in parentheses denote the number of LHE protein sequences included for each intron family. Bootstrap support values (percent) are shown at internal branches, and branch lengths are proportional to the number of substitutions per site (scale bar shown). Two major LHE clades (LHE1 and LHE2) are delineated based on phylogenetic topology and distinct structural features observed in the LHE motifs (colored brackets). The LHEs of the *cox1*-874 intron family are split into two subgroups (*cox1*-874a and *cox1*-874b) according to the phylogenetic topology. Sequence logos to the right summarize the conserved LAGLIDADG regions for each intron family. Motif 1 and Motif 2 are shown for families encoding typical double-motif LHEs, whereas families encoding single-motif LHEs are indicated as having only one motif. The corresponding intron RNA backbone types (IIA1, IIB1, IIB2, and IIC) associated with each LHE lineage are indicated by colored bars on the right. This incongruence provides independent evidence for modular evolution.

Phylogenetic analyses of LHEs encoded by group II-LHE introns revealed two deeply divergent protein lineages (Fig. 7 and Fig. S6). The first lineage (LHE1) includes all LHEs from the five green algal intron families and is subdivided into two clades corresponding to IIB1 and IIB2 RNA backbones. The second lineage (LHE2) contains LHEs from all fungal intron families together with the bacterial family *rns*-1396. Within LHE2, LHEs from five fungal families (IIB1 RNA backbone) and the bacterial *rns*-1396 family (IIC RNA backbone) clustered together and formed a sister relationship to the subclade comprising *cox1*-381 and *cox1*-926 (IIA1 RNA backbone).

The deep split between LHE1 and LHE2 mirrors lineage partitioning across major host groups (green algae versus fungi/bacteria). Notably, the major split between LHE1 and LHE2 does not mirror the four RNA backbone clades in a one-to-one manner, further supporting modular assembly of RNA scaffolds and endonuclease modules. Constrained trees enforcing a single origin of LHE1 and LHE2 were also rejected (AU/SH, *P* < 0.05), consistent with deep divergence among LHE modules. This incongruence indicates that the evolutionary histories of intron RNA scaffolds and their associated LHEs are partially independent.

Motif analyses further revealed strong conservation of the LAGLIDADG motifs across families, with motif 1 generally more conserved than motif 2 (Fig. 7). However, distinct motif patterns among major lineages indicate substantial divergence in LHE ancestry. These results demonstrate that group II-LHE introns represent modular genetic elements composed of independently evolving RNA and protein components.

## Discussion

### Structural and Sequence Divergence Among Group II-LHE Introns

Our analyses reveal extensive structural and sequence heterogeneity among group II-LHE introns across green algae, fungi, and bacteria. Rather than forming a single monophyletic lineage, these elements are distributed across four deeply divergent RNA backbone subclasses (IIA1, IIB1, IIB2, and IIC), consistent with established structural classifications (Toor et al. 2001; Liu et al. 2026). This polyphyly at the backbone level contrasts with expectations from strictly linear models of IEP evolution and suggests a more dynamic evolutionary history.

Unlike canonical group II intron IEPs (RT/M), which often show coevolution with their ribozyme scaffolds despite broad phylogenetic conflicts (Zimmerly et al. 2001; Simon et al. 2009), group II-LHE introns display patterns indicative of repeated, independent module acquisition. Previous studies have interpreted embedded LHE ORFs within group II introns as independent invasions or replacements rather than inheritance from a single ancestral composite element (Toor and Zimmerly 2002; Vitorello et al. 2009; Mullineux et al. 2010). Such independent insertions are consistent with the well-documented mobility and horizontal transfer capacity of LHE genes across introns, inteins, and other genomic compartments (Chevalier and Stoddard 2001; Stoddard 2005).

Beyond backbone divergence, several intron families exhibit recurrent loss or degeneration of the DVI branch-point adenosine, a key determinant of canonical lariat-based splicing (Chu et al. 2001; Plangger et al. 2019). This structural alteration is often accompanied by extension of the 5′ terminal linker and partial erosion of EBS2 or other exon-recognition elements (Toor et al. 2006), reflecting substantial departures from canonical group II intron architecture and suggesting relaxed structural constraints (Chen and Pyle 2017). Furthermore, LHE ORFs occupy heterogeneous positions within intron RNA scaffolds (DIB, DIII, and DIV), supporting repeated independent integration (Toor and Zimmerly 2002).

These observations highlight pronounced structural plasticity at both the RNA and protein levels. They underscore a modular evolutionary framework in which conserved intron cores serve as flexible scaffolds for the recurrent recruitment, loss, and replacement of LHE modules. While our results strongly support multiple independent acquisitions based on phylogenetic and structural evidence, uneven taxonomic sampling and database bias may still influence the apparent lineage distribution and should be considered when interpreting lineage-specific enrichment.

### Implications for Splicing Chemistry: Branching Versus Hydrolytic Pathways

The recurrent structural modifications observed in group II-LHE introns have important implications for splicing chemistry. In several IIB1-LHE families, loss of the DVI branch-point adenosine and extension of the 5′ terminal linker may reflect a shift away from canonical lariat formation and, in some cases, toward hydrolytic splicing (Lambowitz and Zimmerly 2011). Hydrolytic pathways are well documented in structurally reduced group IIC introns and in diverse organellar systems (Toor et al. 2006). For example, in plants, excised introns lacking a DVI bulged adenosine can be released as linear molecules consistent with hydrolysis (Vogel and Börner 2002; Li-Pook-Than and Bonen 2006). However, lariat-based splicing has also been experimentally demonstrated for some IIB1 LHE-encoding introns, notably the mitochondrial *rns*-952 intron (Mullineux et al. 2010), indicating that loss or degeneration of canonical DVI features does not necessarily preclude branching. Alternative pathways such as circularization have also been reported for some group II introns in plants and bacteria (Li and Jiang 2024). These observations suggest that the relative contributions of branching, hydrolysis, and circularization may differ among lineages.

These findings indicate that the altered structures of group II-LHE introns should not be interpreted simply as nonfunctional intermediates; rather, they may remain compatible with multiple splicing chemistries that relax strict dependence on a conserved branch-point architecture (Vogel and Börner 2002; Chan et al. 2025). Such flexibility may facilitate the long-term persistence of ribozyme cores despite extensive peripheral remodeling (Zimmerly and Semper 2015). Importantly, these structural adaptations may have enabled repeated acquisition of LHE modules onto intron scaffolds less dependent on RT/M-mediated retrohoming. By reducing reliance on RNA-based mobility, introns could exploit DNA-level homing mediated by LHEs (Haack et al. 2019), thereby complementing or replacing canonical retrohoming pathways (Lambowitz and Zimmerly 2004; Stoddard 2011, 2014).

Overall, the diversity of splicing chemistries further underscores the evolutionary plasticity of group II introns and supports the view that structural remodeling of the RNA scaffold can facilitate lineage-specific recruitment of LHE modules.

### Modular Assembly and Potential Origins of Group II-LHE Introns

The structural and phylogenetic patterns observed in this study indicate that the evolution of group II-LHE introns is best explained by recurrent modular assembly rather than descent from a single ancestral intron-associated protein lineage (Zimmerly and Semper 2015). LHEs occur on multiple, deeply divergent group II intron backbones, indicating independent recruitment onto distinct ribozyme scaffolds (Mullineux et al. 2010; Megarioti and Kouvelis 2020). This contrasts with linear models in which canonical RT/M-containing group II introns progressively lose retrotransposition capacity before acquiring an LHE (Lambowitz and Zimmerly 2011). Notably, all group II-LHE introns analyzed here lack recognizable RT/M-type IEPs, suggesting that retrohoming-based mobility was lost prior to LHE acquisition. In this context, LHEs provide an alternative mechanism for DNA-level intron propagation, acting as interchangeable mobility modules rather than a terminal evolutionary stage (Stoddard 2011, 2014).

The modular nature of these introns is further supported by the existence of two major LHE lineages, unevenly distributed across RNA backbone subclasses, and by frequent truncated or degenerated ORFs (Megarioti and Kouvelis 2020). These patterns reflect recurring cycles of invasion, fixation, degeneration, and replacement characteristic of selfish genetic elements under fluctuating selective pressures (Belfort et al. 2002; Gogarten and Hilario 2006; Stoddard 2014). BLASTP analyses indicate that most LHEs are closely related to homing endonucleases encoded by group I introns. This pattern was consistent across major LHE lineages and supports the hypothesis that LHE modules in group II-LHE introns were primarily recruited from the broader pool of group I intron–encoded homing endonucleases. The LHEs in the green algal *rnl*-2698/*rns*-420 and *rnl*-2080/*rns*-670 families resemble those in group IB introns, *cox1*-734 and *cox1*-281, respectively (Liu and Melton 2021), and the fungal *rnl*-1787 LHE is closely related to that in the group IB intron (*rnl*-1923) (Sethuraman et al. 2013).

These observations suggest that group II introns may have captured LHE modules originally encoded by group I introns via recombination or horizontal gene transfer. LHE genes could have been mobilized into pre-existing group II intron scaffolds, particularly within structurally permissive regions. Such cross-class recruitment is consistent with the well-documented mobility and modular exchange of homing endonucleases across introns, inteins, and other mobile genetic elements (Gogarten and Hilario 2006; Zubaer et al. 2018). This scenario implies that group II-LHE introns are chimeric elements, in which conserved ribozyme cores provide catalytic splicing activity while LHEs contribute an alternative mobility module capable of DNA-level homing. Recurrent recruitment from the broader organellar LHE pool explains both the heterogeneous insertion sites of LHE ORFs and the deep phylogenetic divergence observed among LHE lineages.

Although a single-origin scenario cannot be formally excluded, it would require repeated convergent restructuring of divergent RNA backbones, independent shifts in ORF insertion sites, and widespread erosion of RT/M domains across unrelated lineages (Zimmerly and Semper 2015). In contrast, RNA backbone polyphyly, incongruence between RNA and LHE phylogenies, lineage-restricted family distributions, and rejection of RNA monophyly in topology tests collectively support recurrent, independent recruitment as the dominant evolutionary mechanism shaping group II-LHE introns.

### Factors Underlying the Mitochondrial Confinement of Group II-LHE Introns

A striking feature of group II-LHE introns is their strong confinement to mitochondrial genomes. With the exception of a single bacterial lineage, nearly all known representatives occur in mitochondrial genes of fungi and green algae. Several factors likely contribute to this lineage-specific distribution.

Mitochondrial genomes experience relatively relaxed selective constraints compared with nuclear genomes, facilitating the accumulation and long-term persistence of mobile introns and other selfish genetic elements. Intron-rich loci such as *cox1*, *rnl*, and *rns* tolerate insertions more readily, providing favorable genomic niches for the establishment and maintenance of intron-associated ORFs (Megarioti and Kouvelis 2020; Galindo et al. 2023). Indeed, mitochondrial group II introns can proliferate rapidly and contribute substantially to genome size variation when constraints are reduced (Kim et al. 2022).

Mitochondrial genomes are subject to frequent recombination and intragenomic rearrangements, processes that can enhance intron mobility and the spread of homing endonucleases. By generating site-specific double-strand breaks, LHEs promote recombination-mediated propagation, further supporting the retention and diversification of group II-LHE introns in organellar genomes (Novikova and Belfort 2017; Kouvelis and Hausner 2022). Mitochondrial genomes of fungi and algae often harbor abundant introns with associated ORFs encoding mobility factors, reflecting these dynamics (Megarioti and Kouvelis 2020; Liu et al. 2022a).

Mitochondria serve as long-standing reservoirs of diverse intron types, including both group I and group II introns (e.g. Zubaer et al. 2018; Liu et al. 2022a; Liu et al. 2022b). This intron-rich environment increases the likelihood of cross-class recruitment of intron-encoded ORFs. Because LHEs are predominantly encoded by group I introns, mitochondria provide opportunities for these endonucleases to be mobilized into group II introns, facilitating repeated recruitment of LHE modules into permissive regions of the RNA scaffold (Novikova and Belfort 2017; Kim et al. 2022). The bacterial *rns*-1396 family may reflect either an independent invasion or horizontal transfer from mitochondria.

These factors suggest that mitochondrial genomes offer an evolutionary context particularly conducive to the origin, persistence, and diversification of group II-LHE introns. In contrast, stronger selective constraints and lower intron densities in nuclear or chloroplast genomes likely limit the establishment and maintenance of such composite RNA–protein elements.

### An Evolutionary Model for the Origin and Diversification of Group II-LHE Introns

**Fig. 8.**
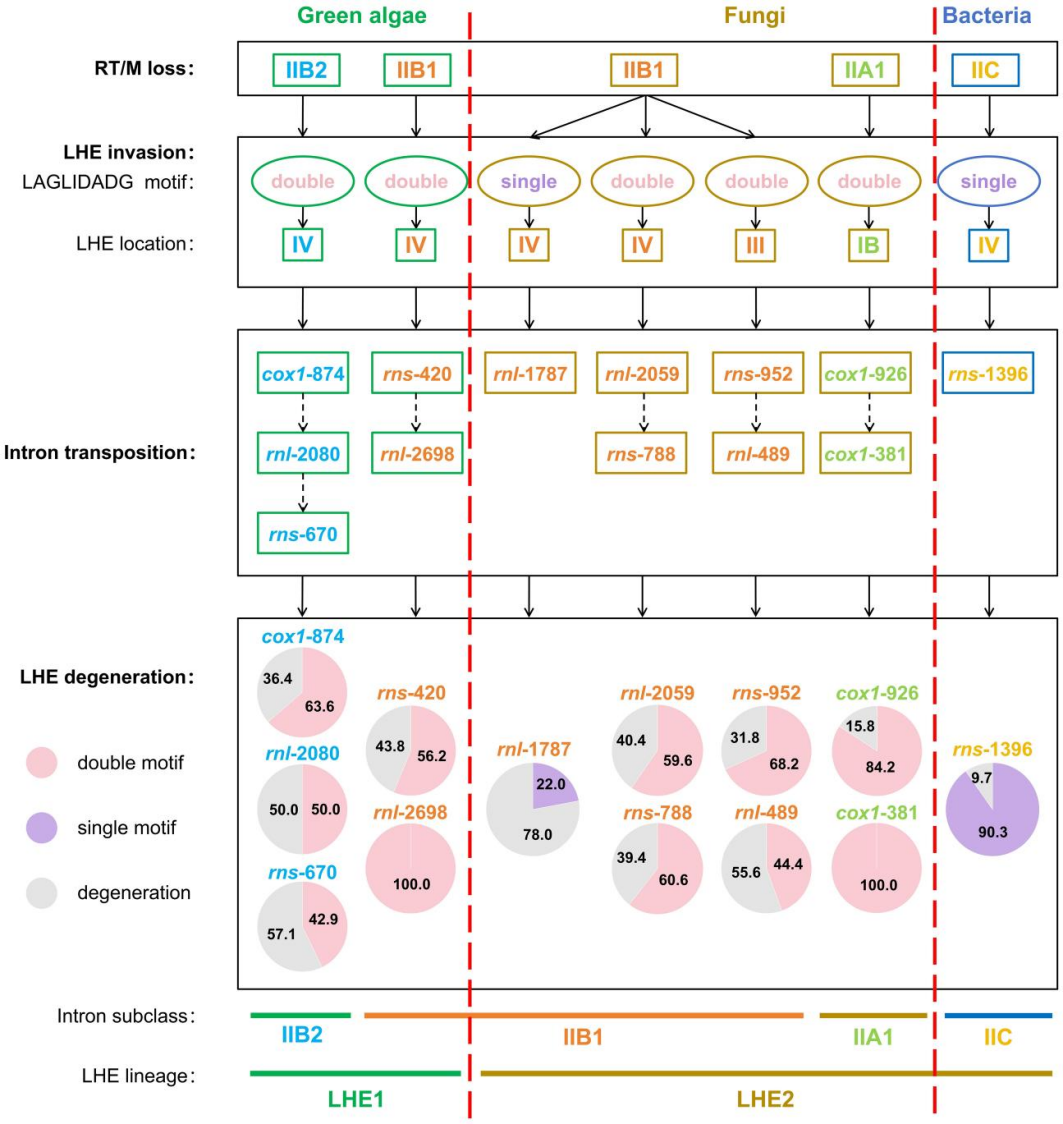
Proposed evolutionary scenario for the origin, diversification, expansion, and degeneration of group II-LHE intron families across major taxonomic groups. Schematic summary integrating the RNA phylogeny, LHE phylogeny, intron family distribution, and structural features to illustrate an evolutionary model for group II-LHE introns in green algae, fungi, and bacteria (lineages separated by red dashed lines). The top panel depicts the inferred loss of the RT/M module, defined here as the complete absence of an RT/M ORF, followed by the invasion of LHEs into the intron RNA scaffold. Boxes indicate the associated group II intron subclass in each lineage (IIB1 and IIB2 in green algae; IIA1 and IIB1 in fungi; IIC in bacteria), LHE motif architecture (double versus single LAGLIDADG motif), and the location of the LHE ORF insertion within well-characterized intron domains (e.g. DIV, DIII, or DIB). The middle panel summarizes the inferred intron transposition events, with dashed arrows indicating possible secondary or derived insertion sites based on RNA phylogeny and structural features. The bottom panel summarizes LHE degeneration within each intron family. Pie charts show the proportion (%) of introns encoding double-motif LHEs, single-motif LHEs, and degenerated LHEs, with percentages labeled. Degenerated LHEs are defined as ORFs exhibiting functional loss due to one or more of the following: premature stop codons, frameshifts, loss of a LAGLIDADG motif, LHE ORF truncation, and/or disruption of key catalytic residues (as detailed in Methods). The bars at the bottom indicate the correspondence between the four intron subclasses (IIA1, IIB1, IIB2, and IIC) and the two major LHE phylogenetic types (LHE1 and LHE2), which are defined based on phylogenetic grouping and structural features observed across intron families.

Integrating intron distribution patterns, RNA structural and phylogenetic classifications, and LHE evolution, we propose that group II-LHE introns originated through recurrent modular invasions of LHE genes into distinct group II intron RNA scaffolds (Fig. 8). In all families examined here, LHE-containing introns lack recognizable RT/M-type IEPs, suggesting that retrohoming-based mobility was lost prior to LHE acquisition. Although the temporal order of RT/M loss and LHE acquisition cannot be resolved directly (Zimmerly and Semper 2015), this mutual exclusivity is more consistent with prior RT/M loss followed by subsequent LHE recruitment than with simultaneous retention of both mobility systems (Lambowitz and Zimmerly 2011; Mayers et al. 2021).

Lineage-specific invasion events are inferred across major clades (Fig. 8). In Ulvales/Ulotrichales, double-motif LHEs were inserted into DIV of IIB1 and IIB2 backbones. In fungi, single- or double-motif LHEs were independently integrated into IIA1 and IIB1 backbones at multiple structural positions (DIB, DIII, and DIV). In bacteria, a single-motif LHE was inserted into DIV of an IIC backbone (*rns*-1396). These patterns collectively support multiple, lineage-restricted recruitment events rather than vertical descent from a single ancestral composite element. Following LHE acquisition, diversification likely involved vertical inheritance, horizontal transfer, and intron transposition (Fig. 8), accompanied by rapid sequence evolution and frequent erosion of targeting determinants such as EBS2, particularly in IIB1 families (Waldern et al. 2020; Mayers et al. 2021). Patterns of sequence similarity and distribution among intron families further support lineage-specific proliferation and differential retention (Mullineux et al. 2010, 2011; Stoddard 2014), while widespread LHE degeneration reflects relaxed selection following mobility loss or functional replacement (Goddard and Burt 1999; Stoddard 2012).

This model accounts for the restricted taxonomic distribution, multiple independent origins, and diverse structural trajectories of group II-LHE introns across green algae, fungi, and bacteria (Fig. 8). More broadly, our findings underscore modularity as a central principle in the evolution of mobile genetic elements (Koonin 2016; Benler and Koonin 2022). The repeated association of LHEs with divergent ribozyme scaffolds illustrates how conserved RNA cores can persist over deep evolutionary timescales while accommodating turnover of associated protein modules (Stoddard 2014; Megarioti and Kouvelis 2020). This modular perspective provides a conceptual framework for understanding the evolution of RNA–protein complexes (Zimmerly and Semper 2015), the origins of spliceosomal introns (Wu et al. 2017; Piccirilli and Staley 2019), and the diversification of RNA-based regulatory systems in eukaryotes (Rebollo et al. 2012; Chuong et al. 2017), highlighting intron evolution as a dynamic process shaped by recurrent recombination and functional reassortment (Werren 2011; Koonin 2016).

## Materials and Methods

### Data Retrieval and Family Naming of Group II-LHE Introns

Group II-LHE introns previously identified in green algae, fungi, and bacteria were used as queries for sequence similarity searches (as of 2026 February 7). The initial query panel consisted of 100 representative sequences, including 66 from green algae, 33 from fungi, and one from bacteria (Table S5). The initial query panel included previously characterized representatives from green algae, fungi, and bacteria, chosen to cover the reported structural and phylogenetic diversity of group II-LHE introns rather than to mirror the taxonomic abundance of currently available genomes. Representative seed sequences were selected from each lineage to provide broad phylogenetic coverage and to reduce lineage-specific retrieval bias during homolog detection. Homologous intron sequences were retrieved from the NCBI GenBank database using BLASTN searches based on conserved group II intron RNA motifs (*E*-value cutoff = 1 × 10^−5^; minimum query coverage = 30%) (Camacho et al. 2009). The relatively relaxed coverage threshold was used to allow detection of divergent, fragmented, or partially degraded intron sequences, including elements lacking intact LHE ORFs but retaining recognizable portions of the conserved intron RNA core. Candidate sequences recovered from similarity searches were then subjected to structural validation.

Sequences were retained only when both of the following core criteria were met: (i) the presence of a recognizable catalytic DV using RNAweasel (Lang et al. 2023), and (ii) a reconstructable group II intron secondary-structure framework spanning domains (DI–DVI). Additional supporting features, when present, included an identifiable EBS1 motif and conserved intron–exon boundary signatures. Sequences lacking DV, or exhibiting such severe fragmentation that reliable reconstruction of the DI–DVI framework was not possible, were excluded from further analysis even if other local motifs were present. In practice, only sequences satisfying both criteria were retained, ensuring consistent and conservative identification across lineages. This screening procedure was designed to prioritize confident identification of bona fide group II-LHE introns while still permitting inclusion of divergent or partially degraded representatives.

In total, we compiled a curated dataset of 753 group II-LHE intron sequences, including 78 mitochondrial introns from the Ulvales/Ulotrichales lineage of green algae, 613 from fungal mitochondrial genomes representing five phyla (Ascomycota, Basidiomycota, Mucoromycota, Blastocladiomycota, and Chytridiomycota), and 62 from bacterial genomes within the Thiotrichaceae family. The final dataset is therefore enriched in fungal sequences. This imbalance may reflect both the greater availability of fungal mitochondrial genomes in current public databases and genuine lineage-specific expansion of group II-LHE introns in fungi. Accordingly, patterns of taxonomic enrichment were interpreted with caution, and evolutionary inferences were evaluated in the context of current database representation.

Intron family determination was primarily based on the insertion site in the homologous host gene and the homology of the entire intron sequence (Liu et al. 2020). Intron families were named using host genes and insertion sites according to established nomenclature conventions (Zhang and Zhang 2019; Liu et al. 2022a). A previously reported intron (*cox1*-686) in *Ulva* sp. (MN853878) (Liu et al. 2026) lacked a recognizable LHE ORF and was excluded from further analysis. Ultimately, we defined 13 distinct group II-LHE intron families. Insertion sites for five green algal introns were determined relative to homologous genes from *Ulva compressa* (KY626327) (Liu et al. 2022a). Previously reported fungal families (*rnl*-1787, *rnl*-2059, *rns*-788, and *rns*-952) and the bacterial family *rns*-1396 were mapped to homologous positions in *Escherichia coli* rRNA genes (AB035922) (Li et al. 2011; Salman et al. 2012; Sethuraman et al. 2013). Newly identified fungal families (*cox1*-381, *cox1*-926, and *rnl*-489) were named based on homologous insertion sites in *Endoconidiophora resinifera* (MK012641).

### RNA Secondary Structure Analysis of Group II-LHE Introns

Secondary structure prediction was performed to reconstruct representative intron structures and key interaction sites. For each intron family, consensus sequences were generated using a majority-rule approach based on multiple sequence alignments. RNA secondary structures were predicted using RNAstructure (Reuter and Mathews 2010) and mfold (Zuker 2003), and structural diagrams were generated using VARNA (Darty et al. 2009). Basic sequence features including total length, domain-specific lengths, and GC content were calculated using custom R scripts. Exon sequences flanking intron insertion sites were aligned in MEGA 11.0 (Tamura et al. 2021) and visualized using WebLogo (Crooks et al. 2004) to examine conserved sequence features associated with intron integration. The LHE ORFs were identified using NCBI ORF Finder, and their positions within intron RNA secondary structures were mapped. SWISS-MODEL was used to assess structural plausibility of representative LHEs and to support classification of single- versus double-LAGLIDADG motif architectures (Waterhouse et al. 2018). Degenerated LHEs were defined as ORFs exhibiting putative functional loss due to one or more of the following features: premature stop codons, frameshifts, loss of a LAGLIDADG motif, LHE ORF truncation, and/or disruption of key catalytic residues. To assess the potential origin of LHE modules, representative LHE sequences were queried against the NCBI GenBank database using BLASTP (*E*-value cutoff = 1 × 10^−5^; minimum query coverage of 30%), and the top hits were classified based on their annotated genomic context.

### Phylogenetic Analyses based on Conserved Intron RNA Sequences and LHEs

To infer evolutionary relationships among group II-LHE intron families, phylogenetic analyses were performed using conserved intron RNA regions. Catalytic core sequences were manually extracted from each intron instance, and mutational hotspot regions were excluded, including the 5′ terminal linker, the EBS2-ID2 region, Ia, ICa, IC2, ID(ii)1, ID(iii), ID(iii)2, ID(iv), and loop regions within domains IA, IB, II, III, IV and VI (Toor et al. 2001). These regions were excluded to minimize structural heterogeneity and alignment ambiguity, thereby improving positional homology and the reliability of downstream phylogenetic inference. Multiple sequence alignments were generated using a secondary-structure–guided strategy. Initial alignments were produced with MAFFT v7 using the L-INS-i algorithm, which incorporates local pairwise alignment information and is suitable for sequences with conserved structural elements (Katoh and Standley 2013).

Alignments were then manually refined in the context of predicted RNA secondary structures to preserve homologous pairing interactions and structural domains (DI–DVI). For comparison, alignments generated using MUSCLE (Edgar 2004) were also examined, and phylogenetic inferences were robust to alignment method. The final alignment of 13 intron families contained 615 conserved nucleotide positions. Maximum-likelihood (ML) phylogenetic trees were reconstructed in IQ-TREE v3.0.1 (Wong et al. 2026), with the best-fitting substitution model (GTR+F+I+G4) selected under the Bayesian Information Criterion (BIC) using ModelFinder (Kalyaanamoorthy et al. 2017). Node support was assessed using 1,000 ultrafast bootstrap replicates (Hoang et al. 2018) and 1,000 SH-like aLRT tests (Guindon et al. 2010). RNA phylogenetic patterns were interpreted in the context of host phylogeny and intron distribution to assess evolutionary processes, such as vertical inheritance, horizontal transfer, transposition, and intron proliferation.

Due to substantial length variation among LHEs, phylogenetic analyses focused on conserved regions spanning the LAGLIDADG motif(s) and the intervening linker region. Amino acid sequences were aligned using MUSCLE as implemented in MEGA 11.0 (Tamura et al. 2021) to produce an alignment of 342 conserved residues. ML phylogenetic trees were reconstructed in IQ-TREE v3.0.1 using the same analytical framework as described above. For the LHE amino acid dataset, the best-fitting substitution model (VT+F+G4) was selected under the BIC using ModelFinder. Node support was assessed using 1,000 ultrafast bootstrap replicates and 1,000 SH-like aLRT tests.

Topological congruence between RNA- and LHE-based phylogenies was assessed to evaluate potential co-evolutionary associations between intron RNA scaffolds and their encoded endonucleases. To evaluate potential long-branch attraction and the impact of missing data, we repeated the RNA phylogeny after excluding the most gap-rich alignment positions and/or the most highly divergent family-level subsets. In parallel, we reconstructed the LHE phylogeny after removing truncated or highly degenerated ORFs and, alternatively, using alignments restricted to conserved regions surrounding the LAGLIDADG motifs. In all sensitivity analyses, the overall topologies of both RNA and LHE phylogenies were robust to exclusion of highly divergent sequences or manual removal of poorly aligned regions, recovering the same major clades observed in the primary analyses (i.e. the four RNA backbone clades and the two major LHE lineages).

### Topology Tests of Alternative Evolutionary Hypotheses

To statistically evaluate competing origin scenarios, we performed topology tests comparing the ML tree inferred without constraints to ML trees inferred under explicit topological constraints, thereby explicitly testing alternative evolutionary scenarios and distinguishing between single-origin and multiple-origin models. For the intron RNA dataset, we generated constrained trees enforcing monophyly of all group II-LHE introns, representing a single-origin hypothesis, and compared them with the best unconstrained topology. Constrained and unconstrained ML trees were inferred in IQ-TREE v3.0.1 (Minh et al. 2020) under the same substitution model used for the primary analyses (GTR+F+I+G4). Site-wise log-likelihoods for each competing topology were computed in IQ-TREE, and tree topologies were evaluated using the approximately unbiased (AU) test (Shimodaira 2002) and Shimodaira–Hasegawa (SH) test (Shimodaira and Hasegawa 1999) with 10,000 RELL replicates. Constrained hypotheses were considered rejected when *P* < 0.05. For the LHE amino acid dataset, we similarly compared the best unconstrained ML topology with constrained trees enforcing alternative monophyly hypotheses (e.g. a single LHE origin), using the same AU/SH framework (VT+F+G4).

## Supplementary Material

evag114_Supplementary_Data

## Data Availability

The original contributions presented in this study are included in the article. Further inquiries can be directed to the corresponding author.
