# Supplementary material for: Recurrent Modular Assembly Drives Independent Origins of Group II Introns Encoding LAGLIDADG Homing Endonucleases
Source: Genome Biol Evol. 2026 May 6;18(5):evag114. doi: 10.1093/gbe/evag114 (PMC13190283; doi:10.1093/gbe/evag114)
Supplement: evag114_Supplementary_Data [file Supplementary Figure S1-S6.pdf › Supplementary Figure S1-S6.pdf]

A. Green algae mt IIB2-LHE *rnl*-2080 (9)

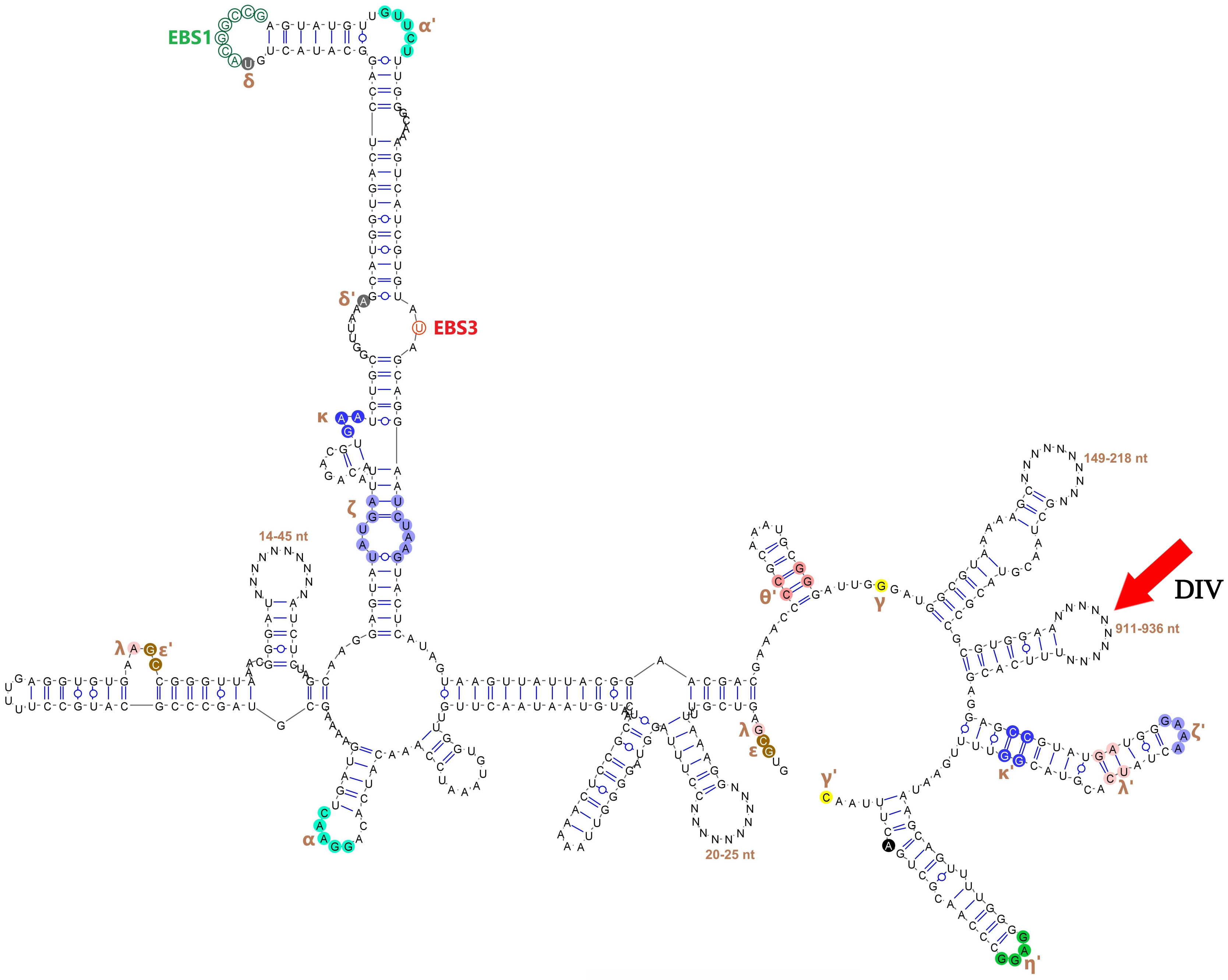

B. Green algae mt IIB1-LHE *rnl*-2698 (10)

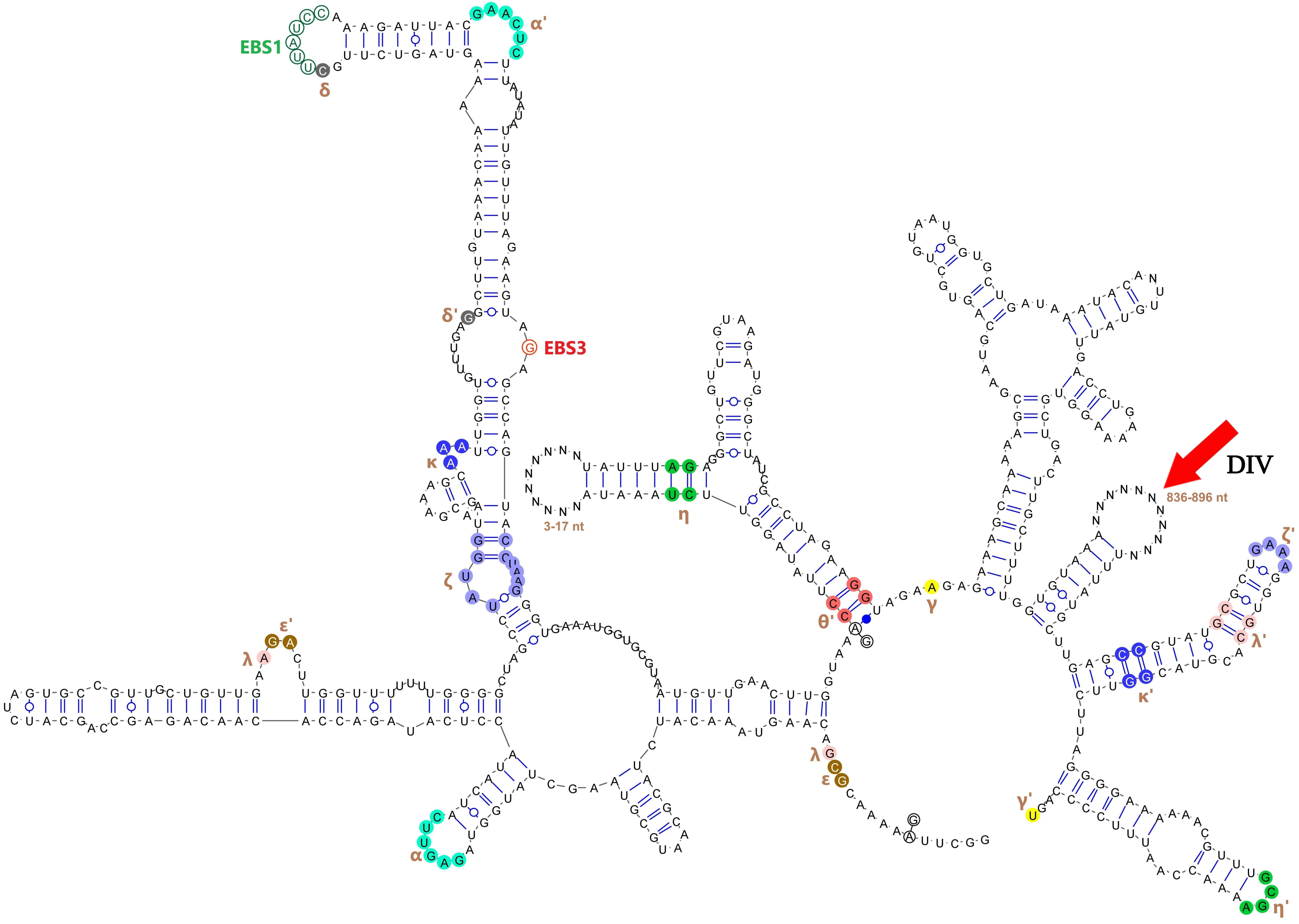

C. Green algae mt IIB1-LHE *rns*-420 with 5' terminal linker (6)

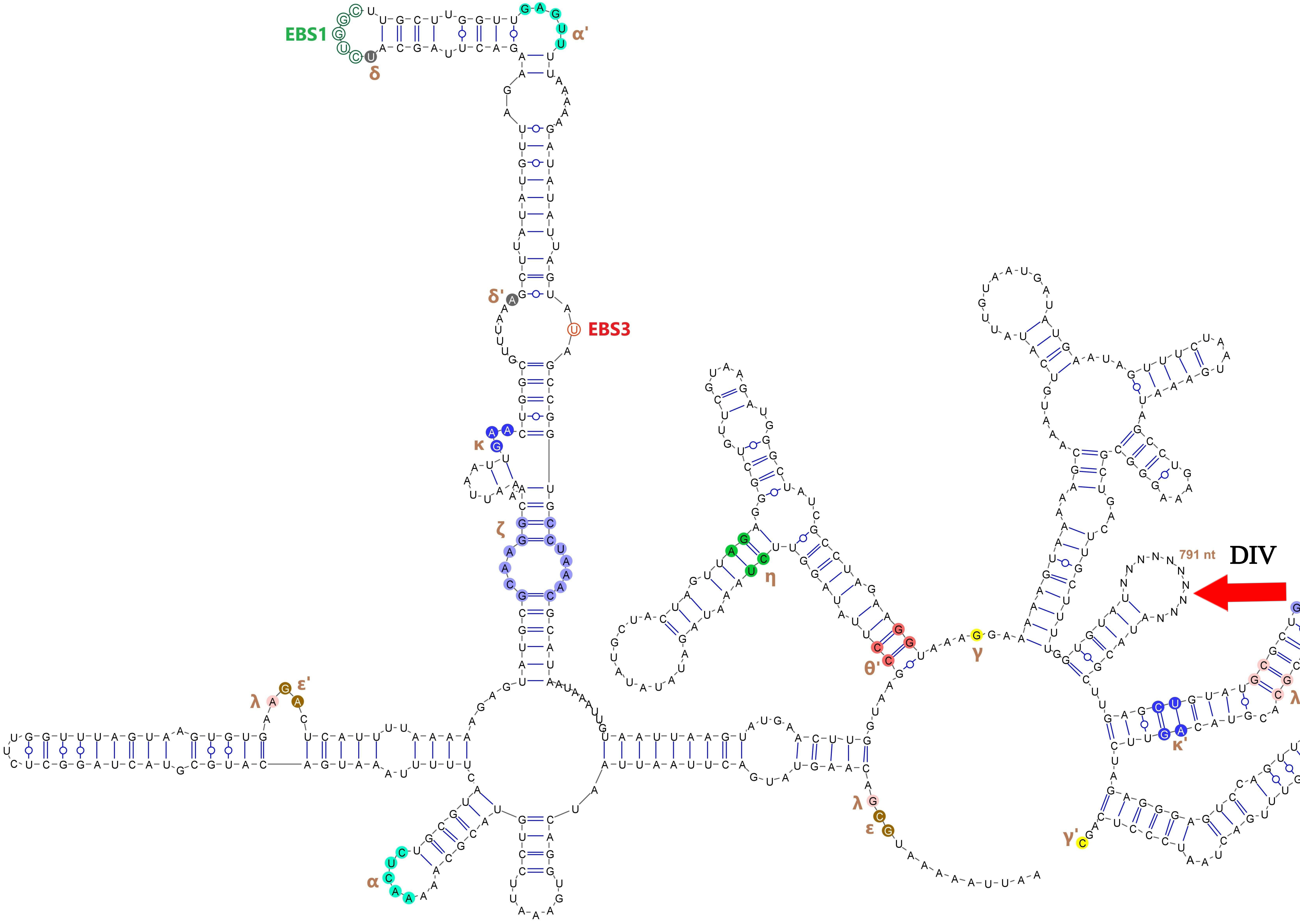

D. Green algae mt IIB1-LHE *rns*-420 without 5' terminal linker (11)

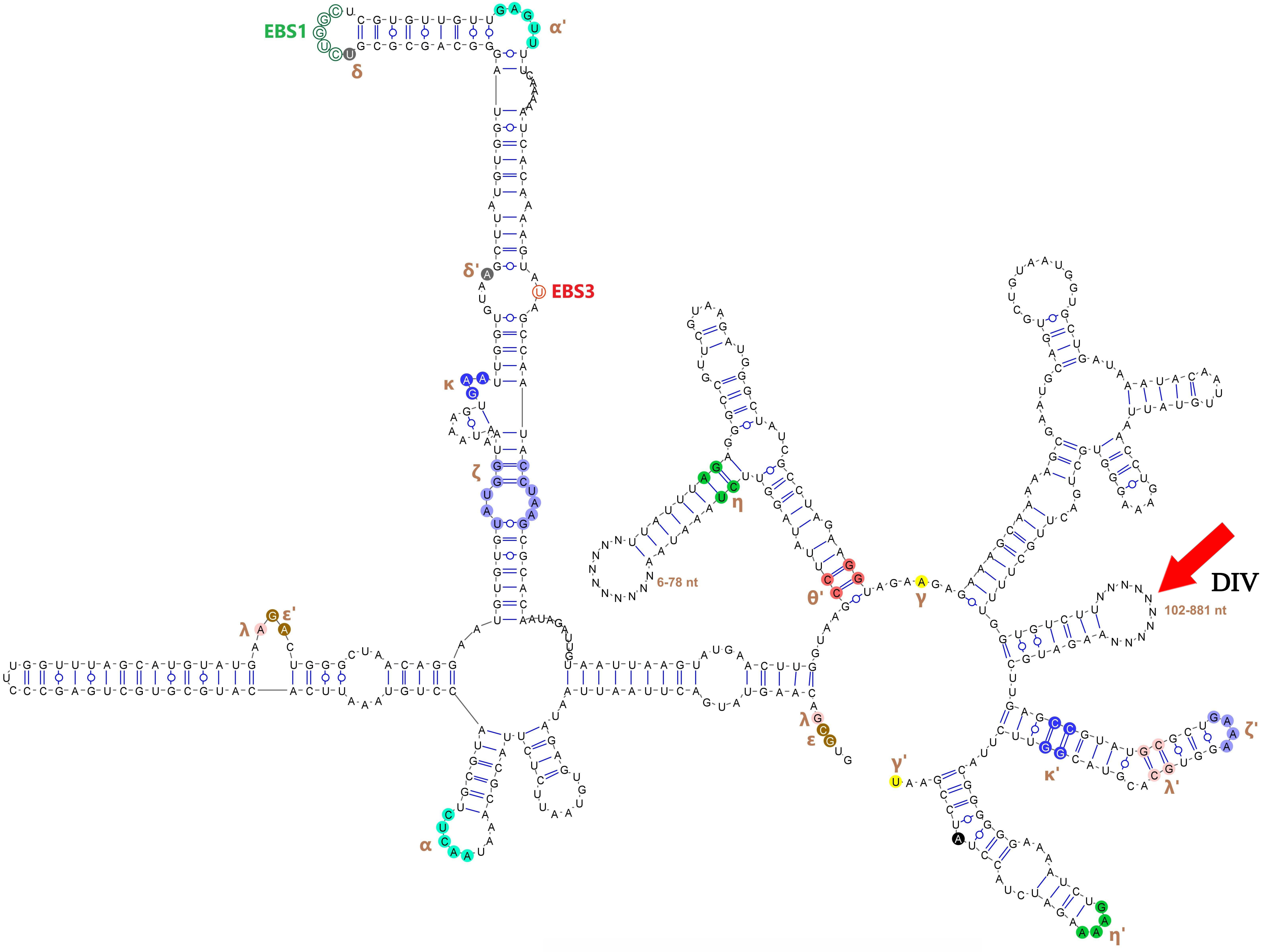

E. Green algae mt IIB2-LHE *rns*-670 (20)

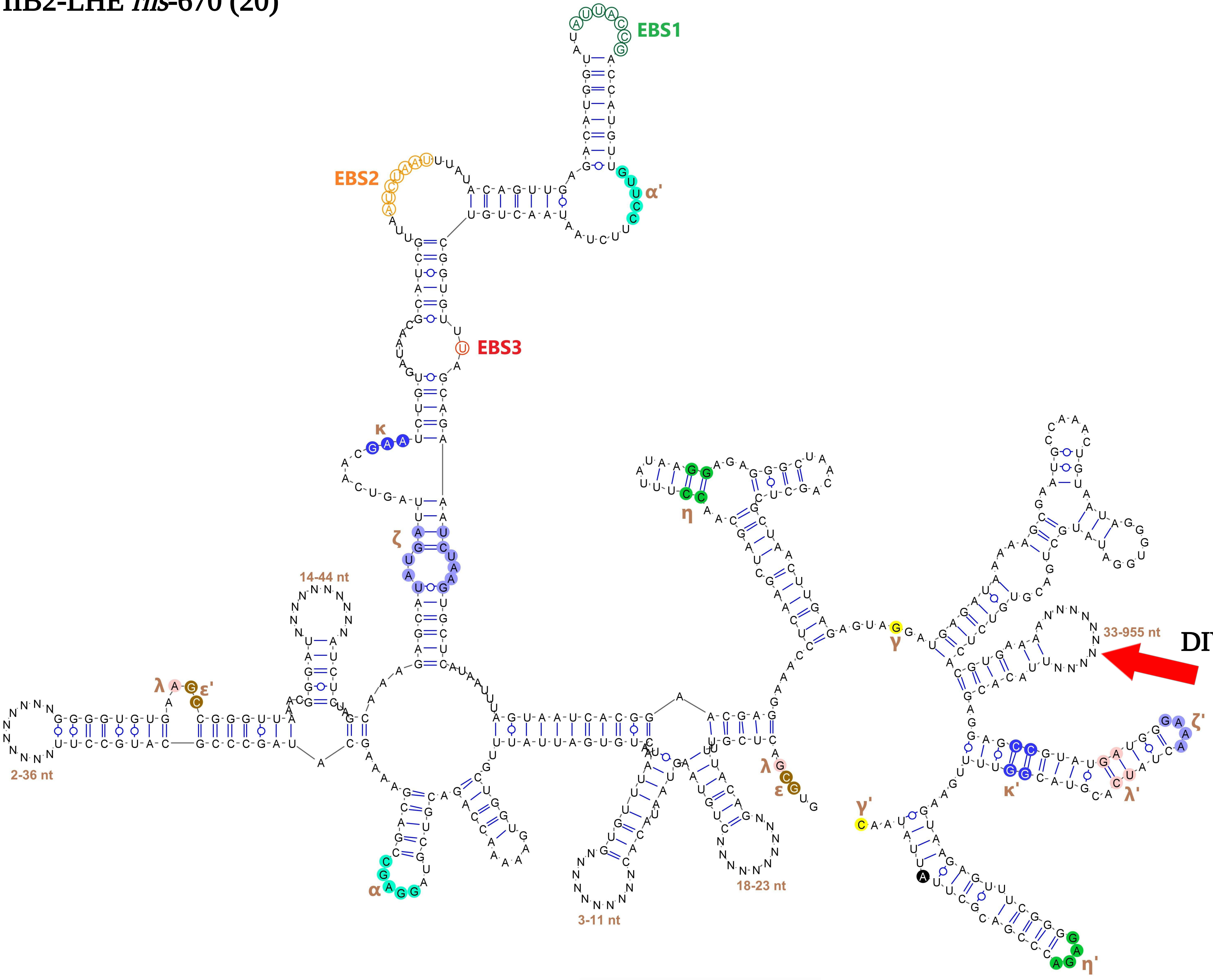

Supplementary Fig. S1 Predicted secondary structures of group II-LHE intron families in green algae. Secondary structure models of representative group II-LHE introns located in mitochondrial genes from green algae are shown, predicted using RNA structure prediction tools (RNAstructure or mfold). Numbers in parentheses indicate group II-LHE intron counts. Conserved structural domains (DI − DVI) are illustrated, with key tertiary interaction motifs (EBS1, EBS2, and EBS3) highlighted. Inserted sequences including LHE ORFs are indicated within variable regions of the introns. Red arrows mark the position of the LHE ORF insertion within the intron RNA scaffold. Nucleotide positions and lengths of insertions are annotated . Distinct colors denote conserved regions and functionally important motifs across different intron types. The diagrams demonstrate structural diversity and conservation among group II-LHE intron families.

A. Fungi mt IIA1-LHE *cox1*-381 (1)
MN317087 *Magnusiomyces paraingens*

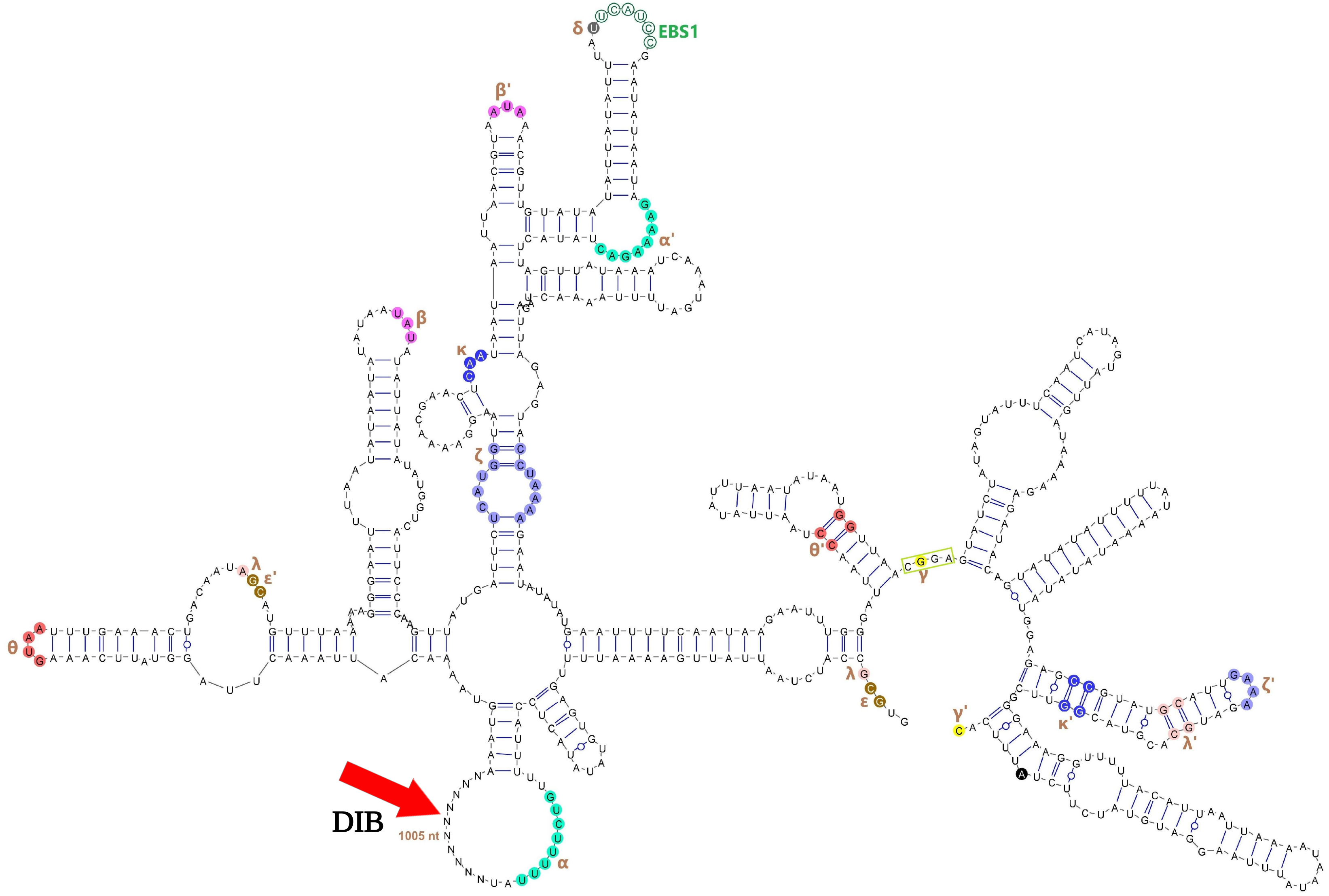

B. Fungi mt IIA1-LHE *cox1*-926 (146)

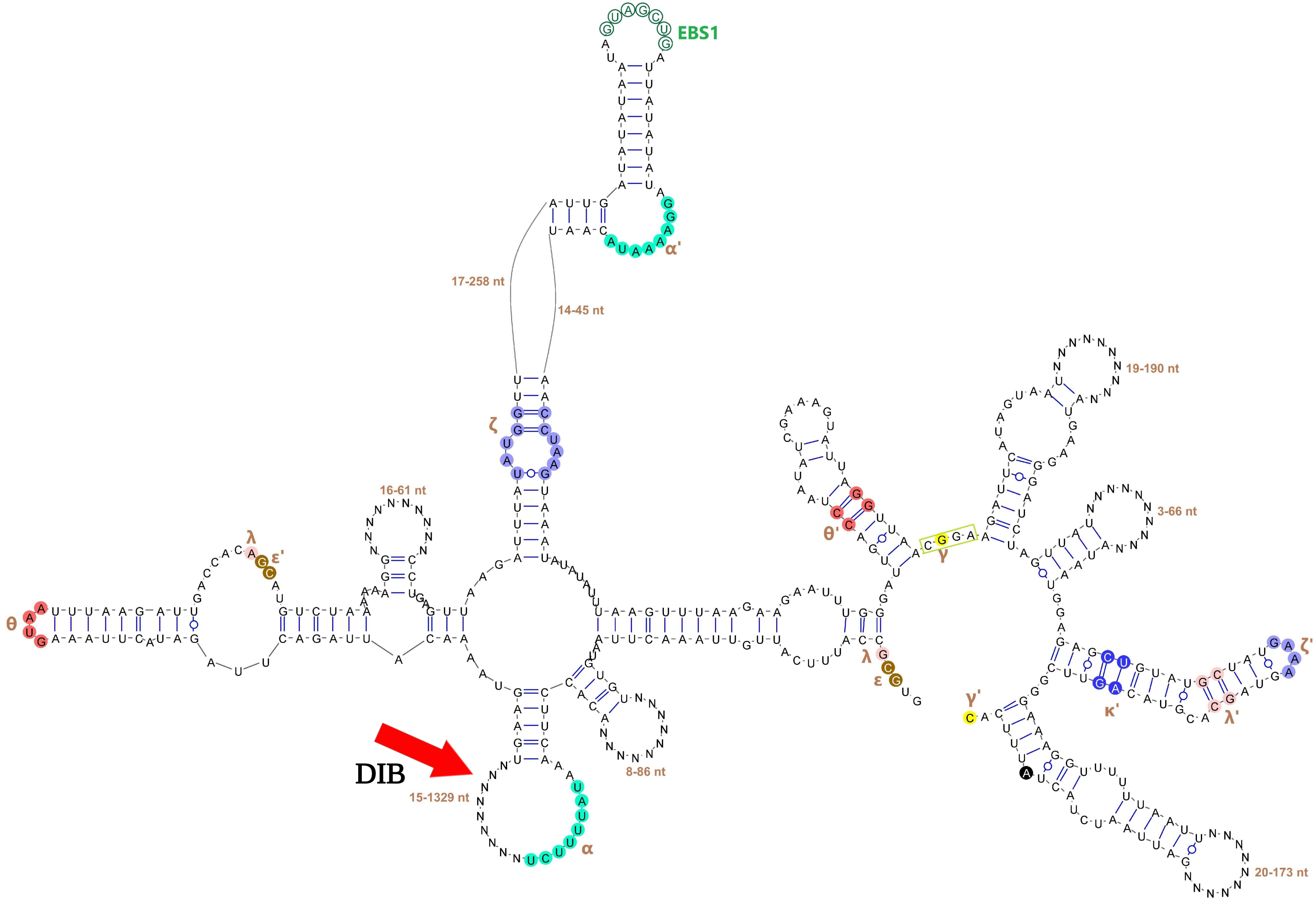

C. Fungi mt IIB1-LHE *rnl*-1787 (59)

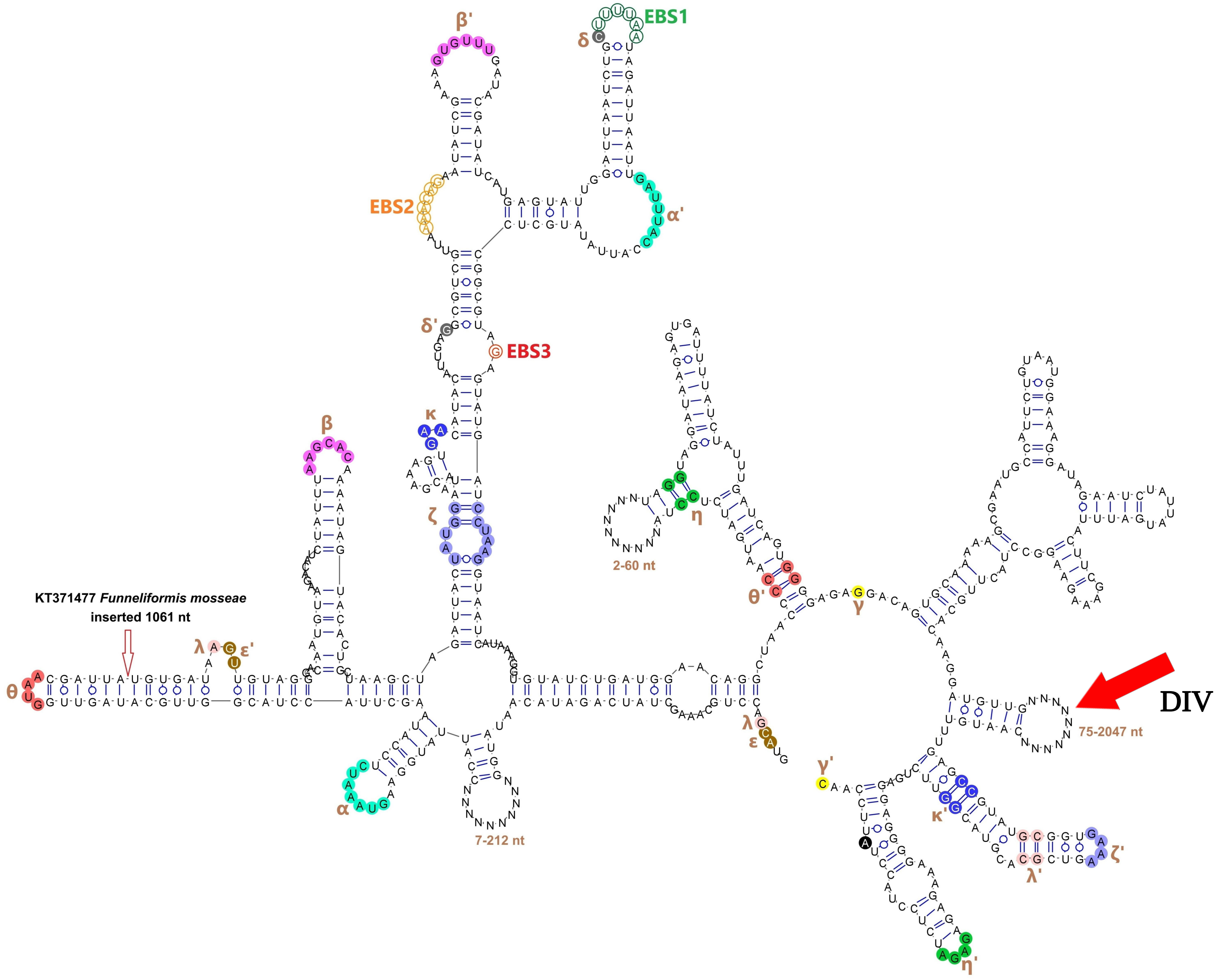

D. Fungi mt IIB1-LHE *rnl*-2059 (1)
U41288 *Allomyces macrogynus*

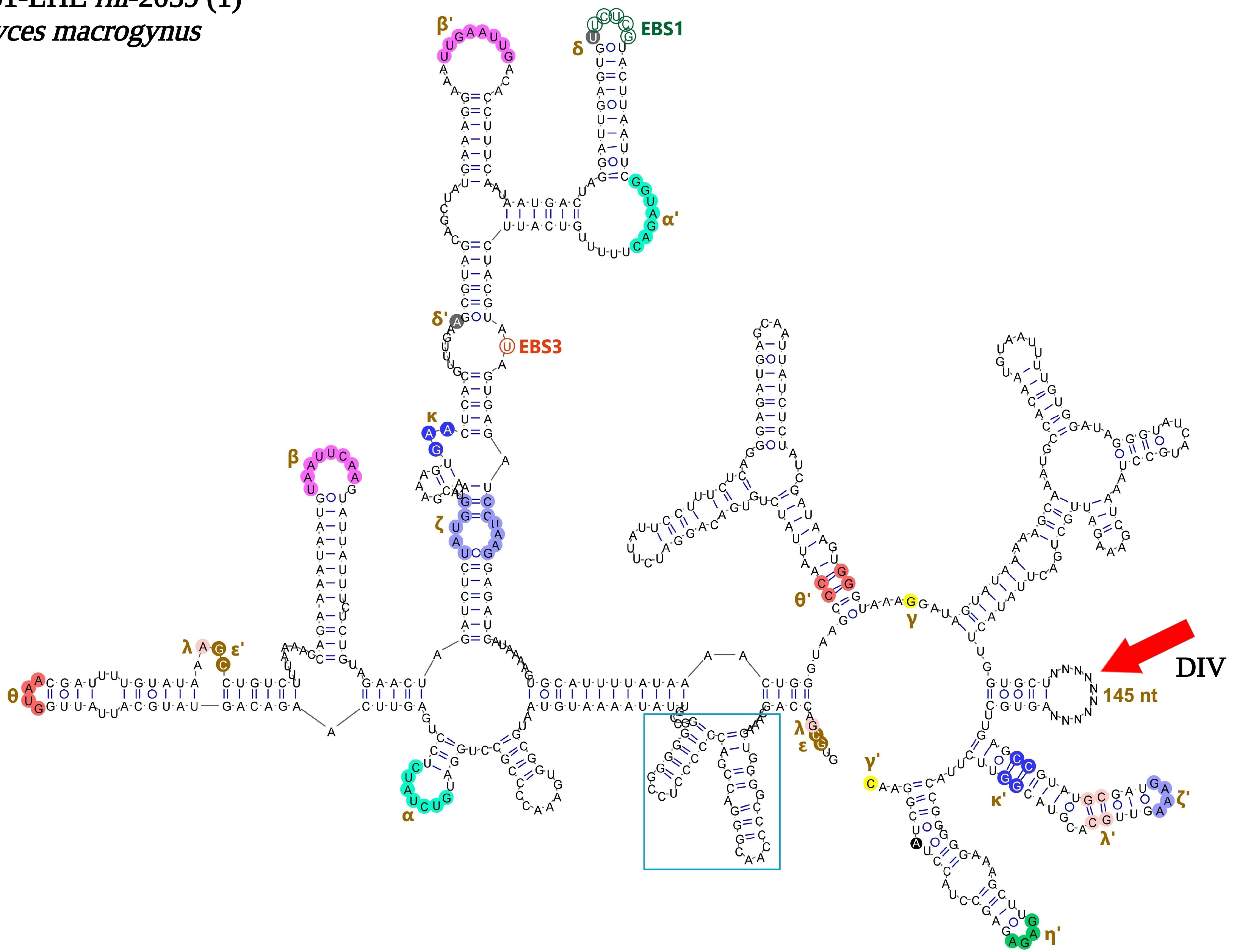

E. Fungi mt IIB1-LHE *rnl*-2059 with 5' terminal linker (111)

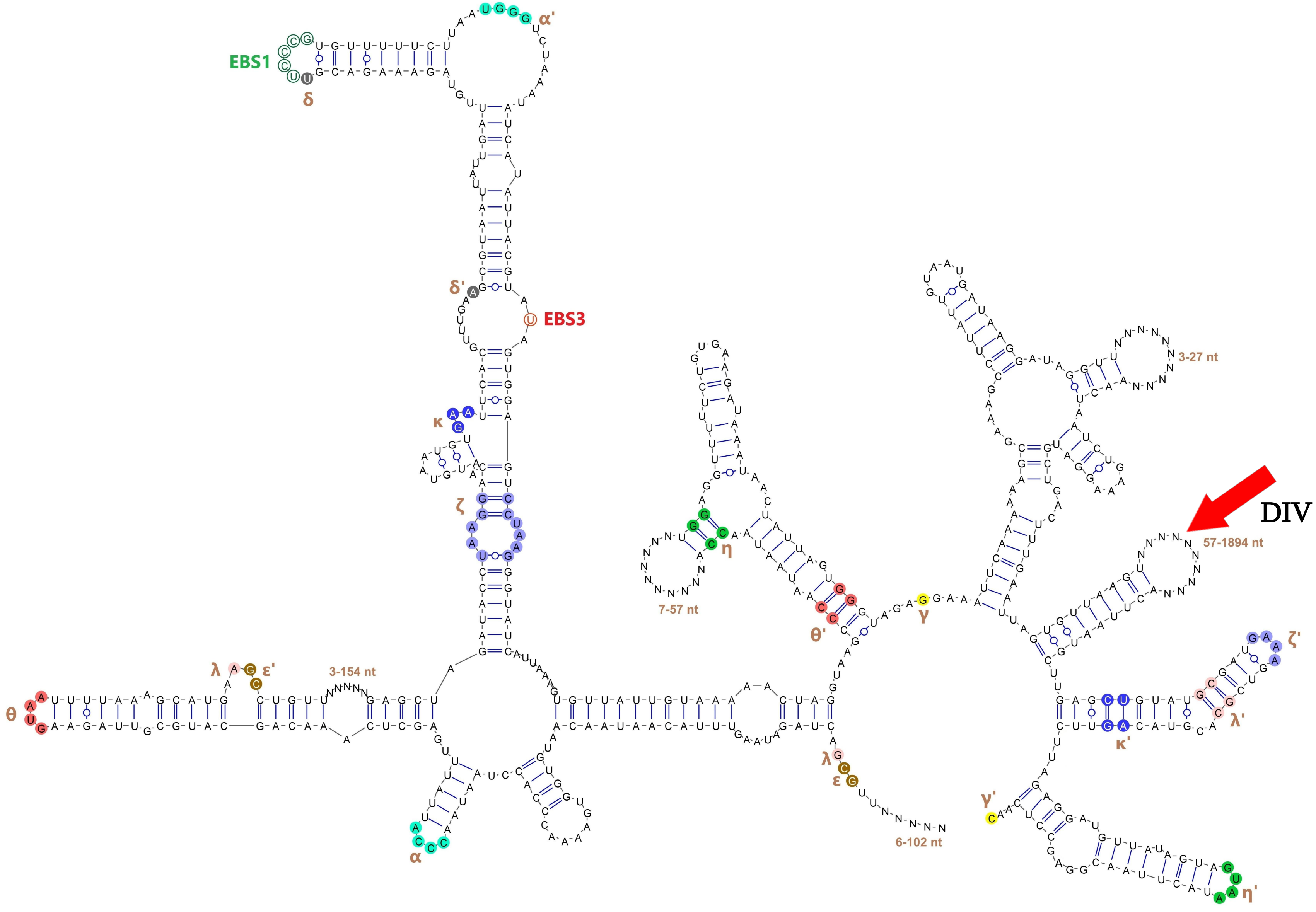

F. Fungi mt IIB1-LHE *rnl*-2059 without 5' terminal linker (97)

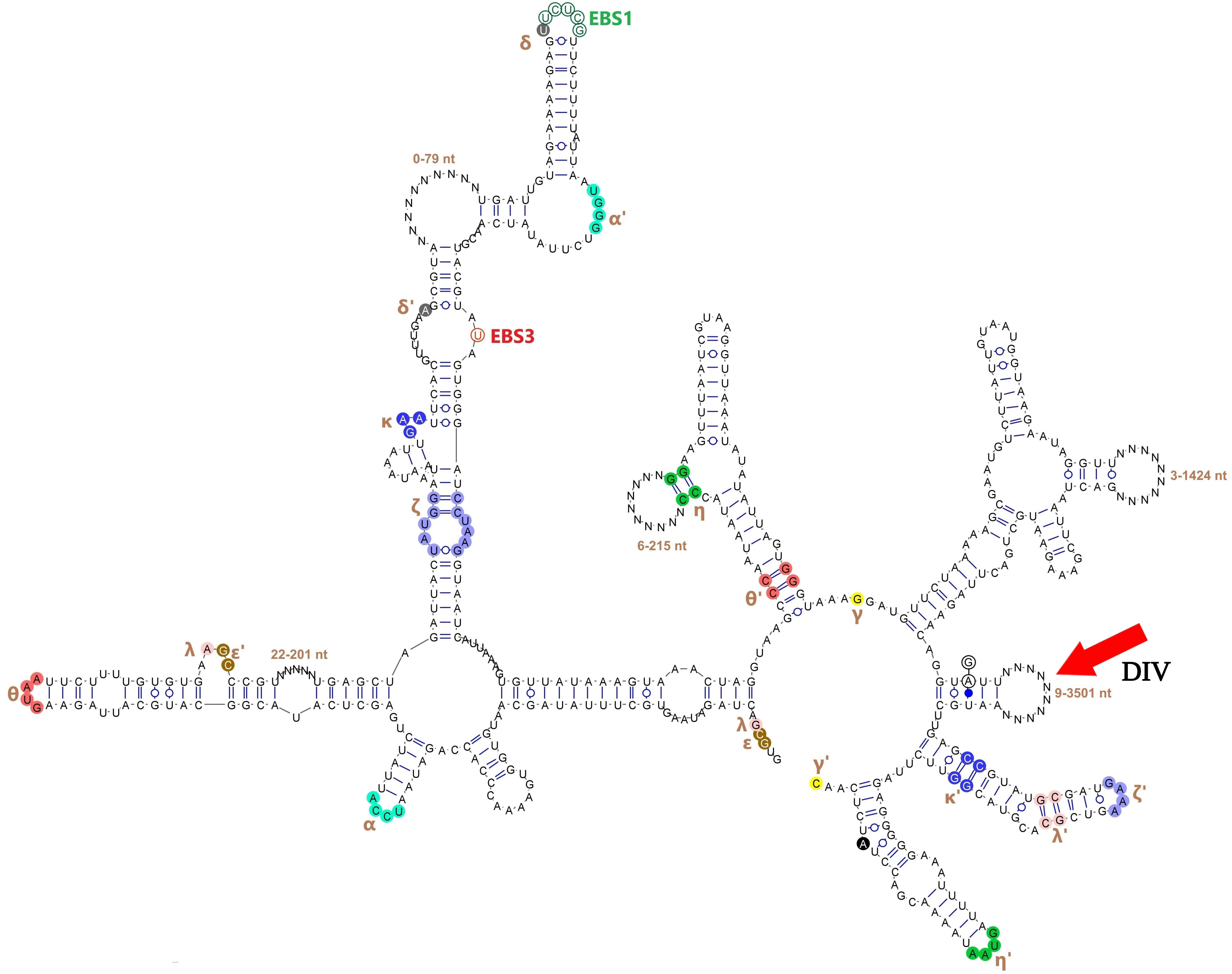

G. Fungi mt IIB1-LHE *rns*-788 with 5' terminal linker (45)

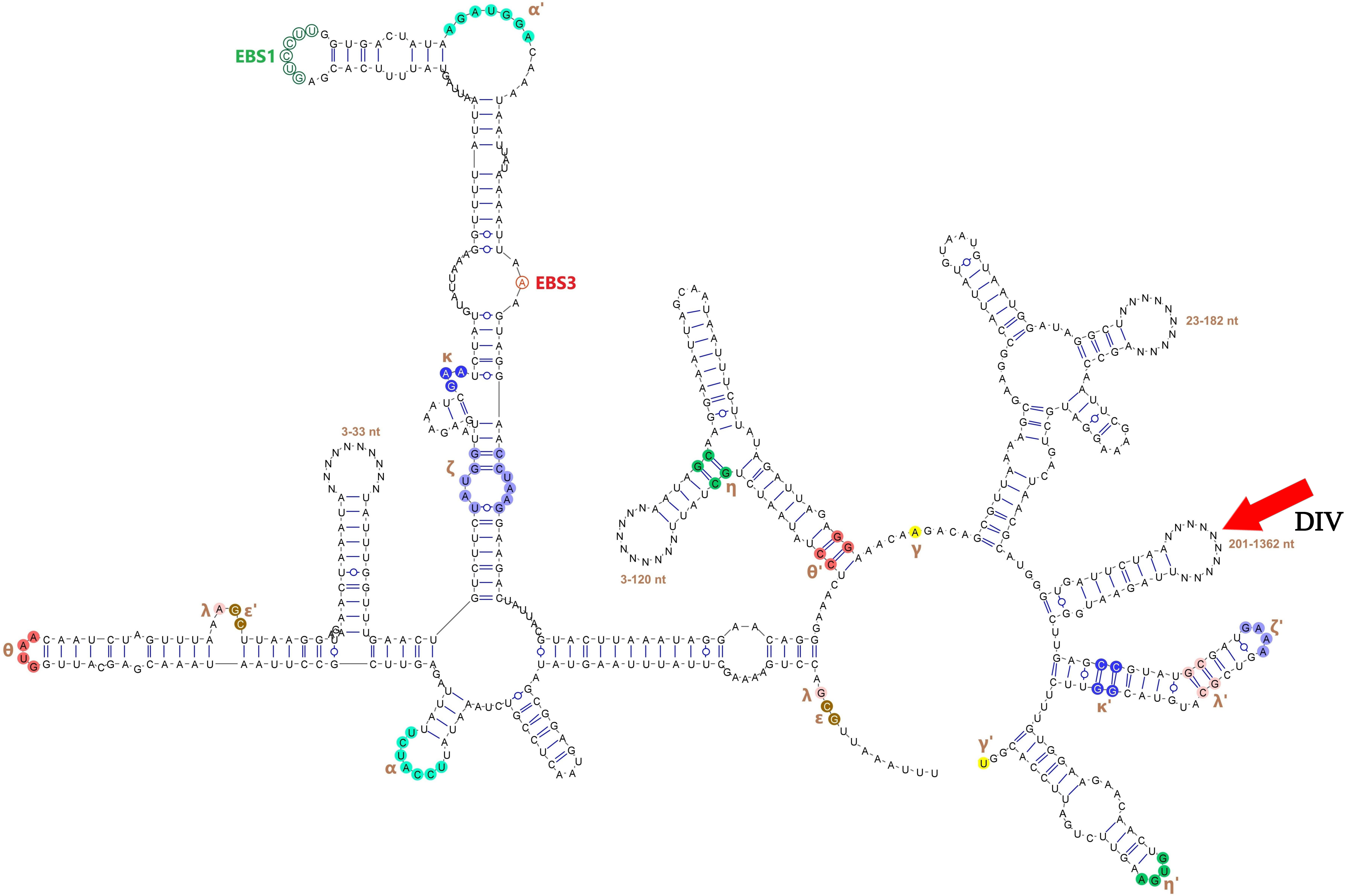

H. Fungi mt IIB1-LHE *rns*-788 without 5' terminal linker (56)

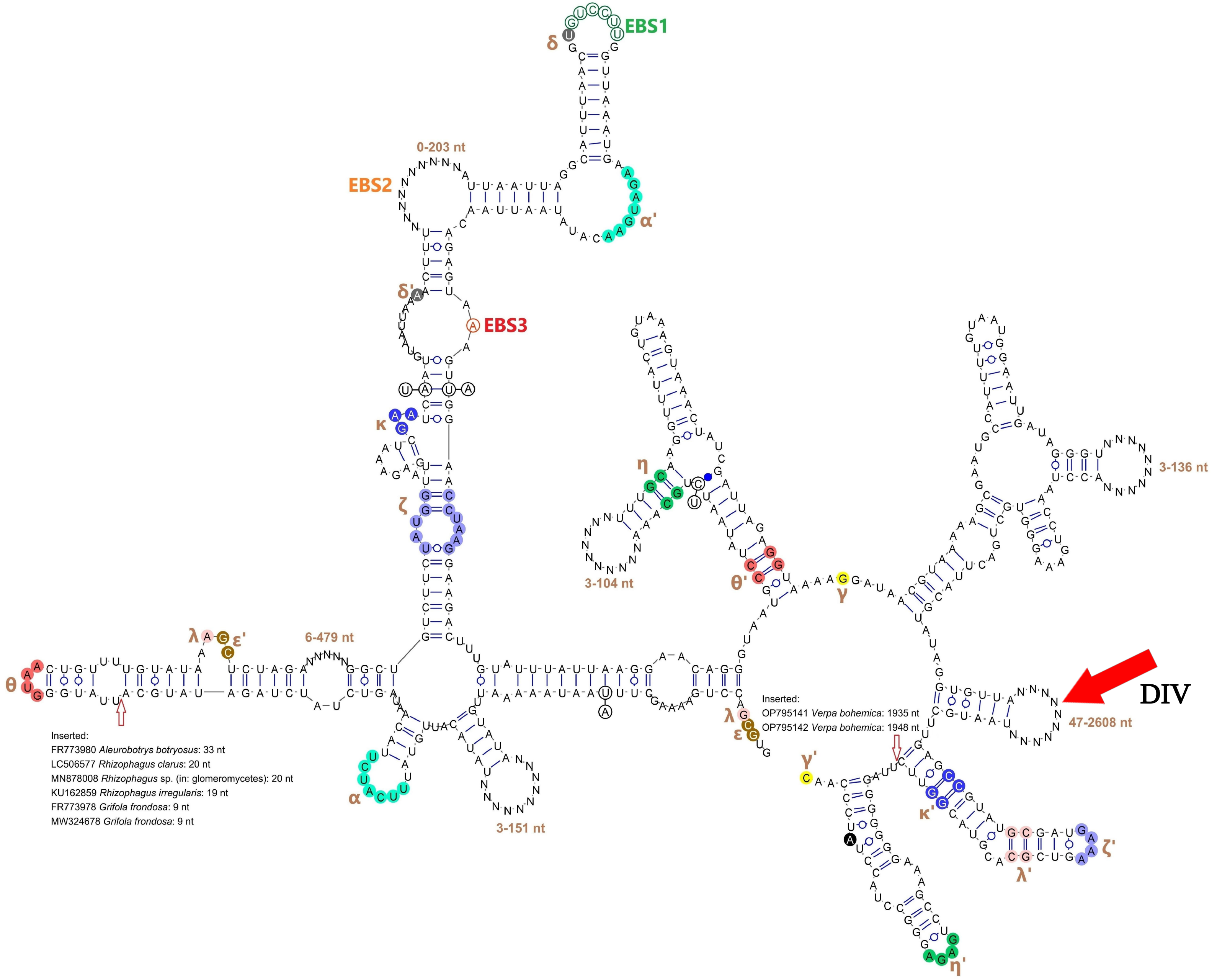

I. Fungi mt IIB1-LHE *rns*-952 (88)

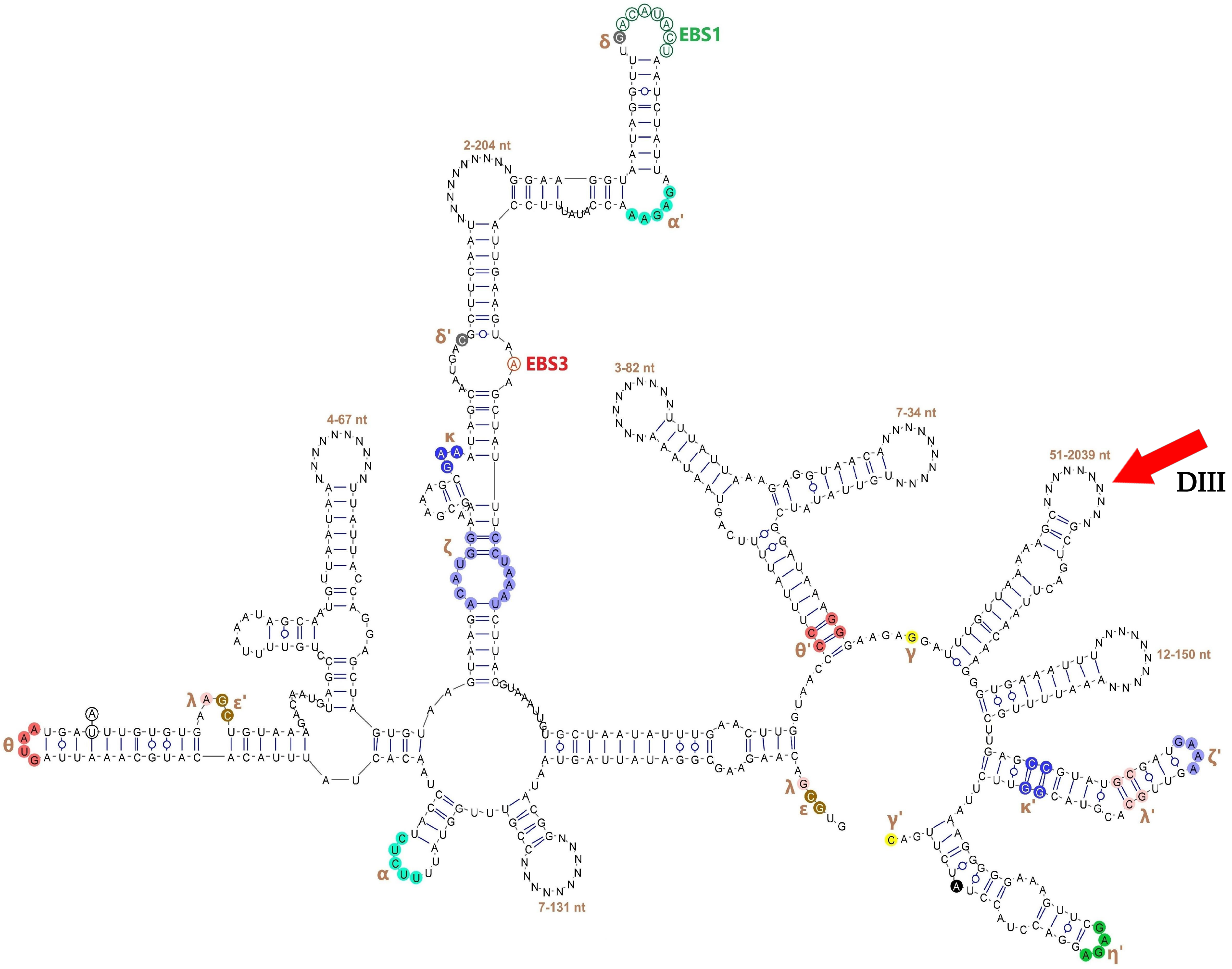

Supplementary Fig. S2 Predicted secondary structures of group II-LHE intron families in fungi. Secondary structure models of representative group II-LHE introns located in mitochondrial genes from fungi, predicted using RNA structure prediction tools (RNAstructure or mfold). Numbers in parentheses indicate group II-LHE intron counts. Conserved structural domains (DI − DVI) are illustrated, with key tertiary interaction motifs (EBS1, EBS2, and EBS3) highlighted. Inserted sequences including LHE ORFs are indicated within variable regions of the introns. Red arrows mark the position of the LHE ORF insertion within the intron RNA scaffold. Nucleotide positions and lengths of insertions are annotated. Distinct colors denote conserved regions and functionally important motifs across different intron types. The diagrams demonstrate structural diversity and conservation among group II-LHE intron families.

A. Bacteria IIC-LHE *rns*-1396 (62)--Version 1

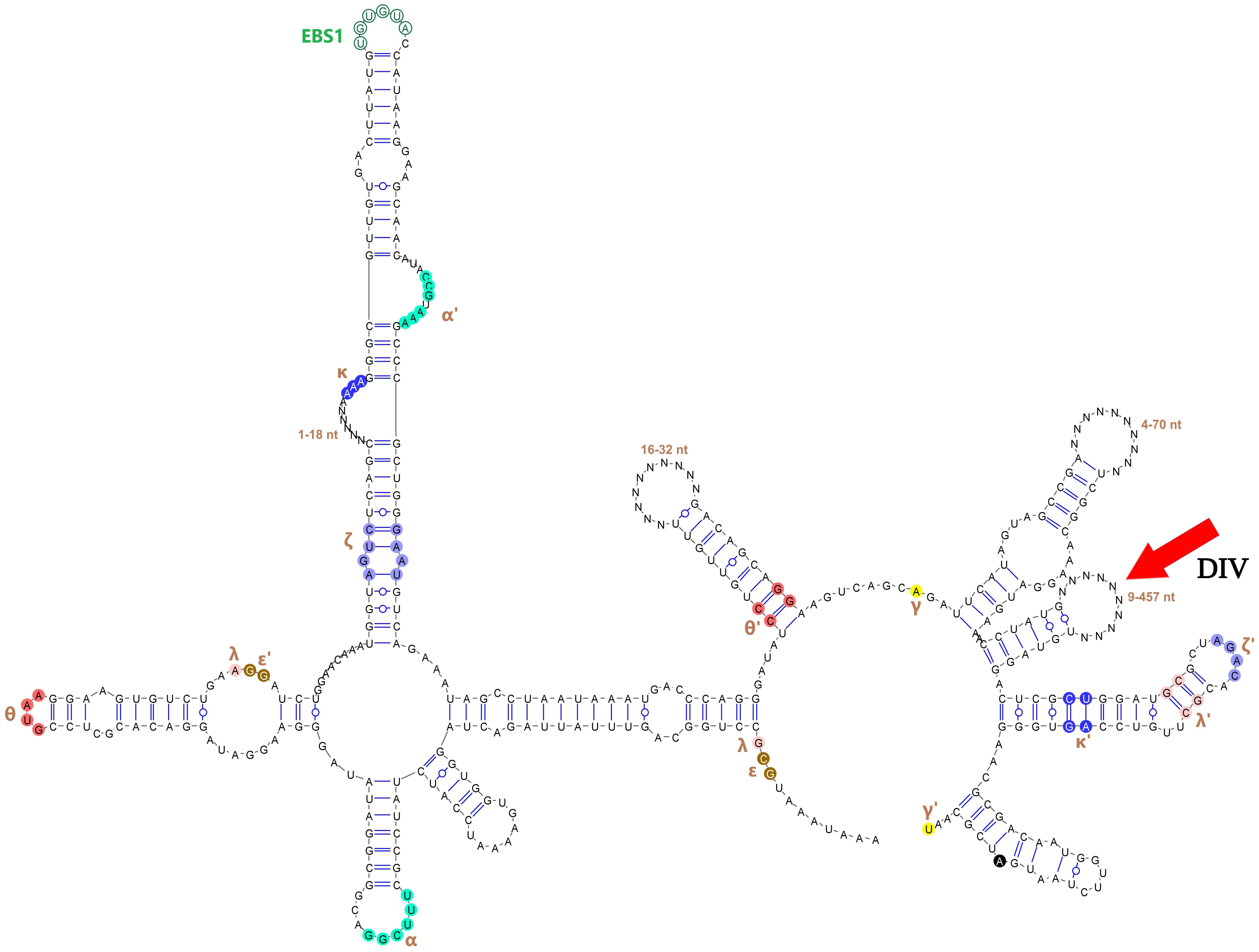

B. Bacteria IIC-LHE *rns*-1396 (62)--Version 2

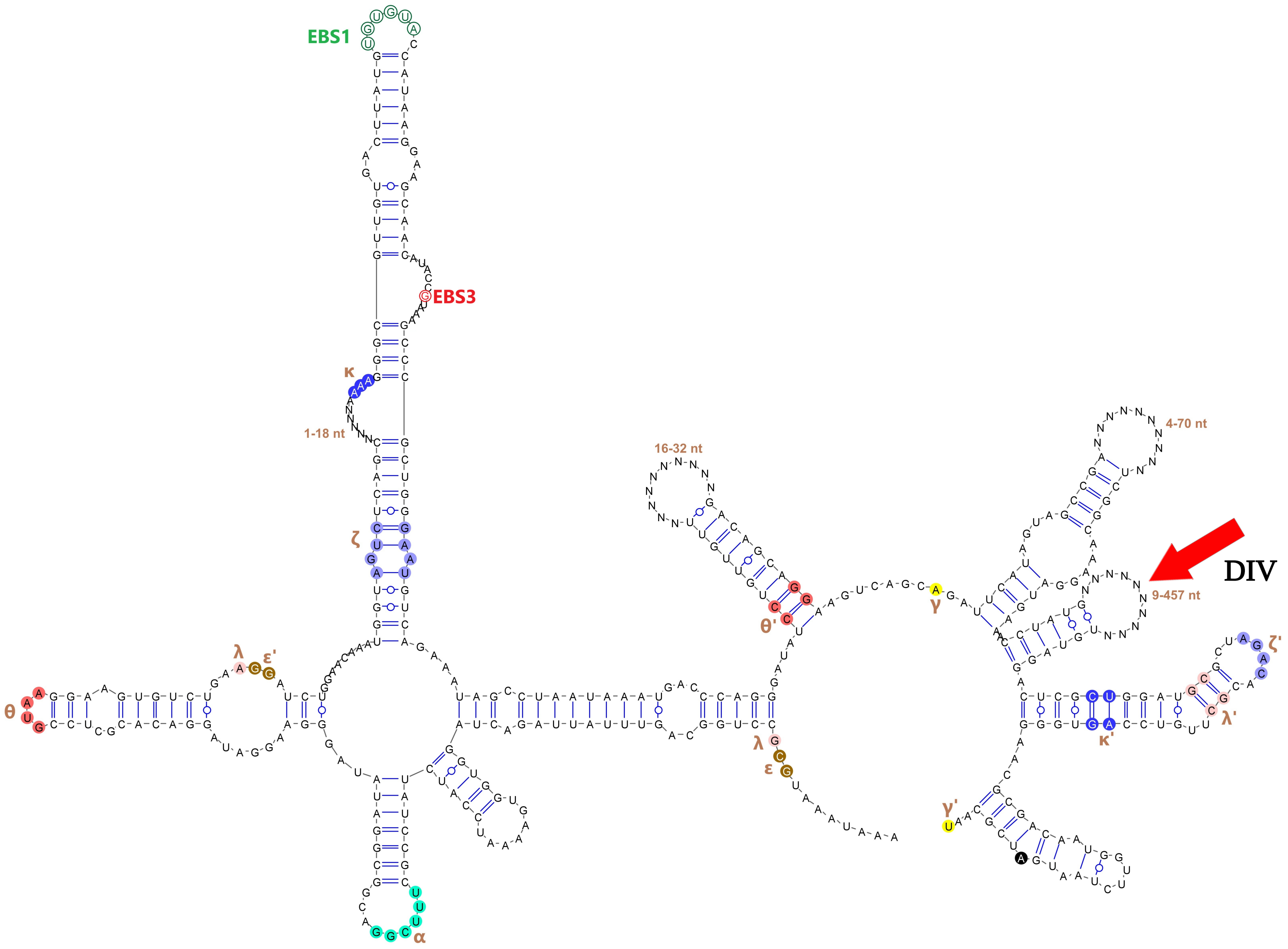

Supplementary Fig. S3 Predicted secondary structures of group II-LHE intron family in bacteria. Two versions of secondary structure models of representative group II-LHE intron family (*rns*-1396) located in *rns* gene from bacteria are shown, predicted using RNA structure prediction tools (RNAstructure or mfold). Numbers in parentheses indicate group II-LHE intron counts. Conserved structural domains (DI − DVI) are illustrated, with key tertiary interaction motifs (EBS1, and EBS3) highlighted. Inserted sequences including LHE ORFs are indicated within variable regions of the introns. Red arrows mark the position of the LHE ORF insertion within the intron RNA scaffold. Nucleotide positions and lengths of insertions are annotated. Distinct colors denote conserved regions and functionally important motifs across different intron types. The diagrams demonstrate structural diversity and conservation among group II-LHE intron families.

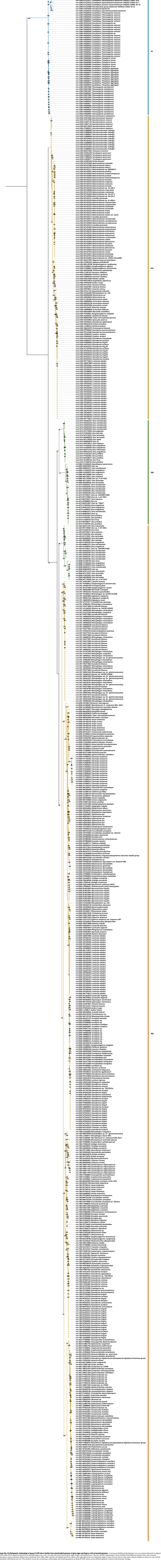

Supplementary Fig. S4 Phylogenetic relationships of group II-LHE intron families from mitochondrial genomes of green algae and fungi as well as bacterial genomes. A maximum-likelihood phylogenetic tree was constructed based on aligned group II-LHE intron RNA sequences derived from multiple genes (e.g., *cox1*, *rnl*, and *rns*) across diverse green algal, fungal, and bacterial taxa. The tree includes representative intron families from different insertion sites with accession numbers and species names indicated. Major intron subclasses (IIA1, IIB1, IIB2, and IIC) are labeled and color-coded on the right side of the tree. Bootstrap support values (%) are shown at the nodes. Branch lengths are drawn to scale and the scale bar represents substitutions per site. This analysis reveals the evolutionary relationships and clustering patterns of group II-LHE introns across different genomic contexts and taxonomic lineages.

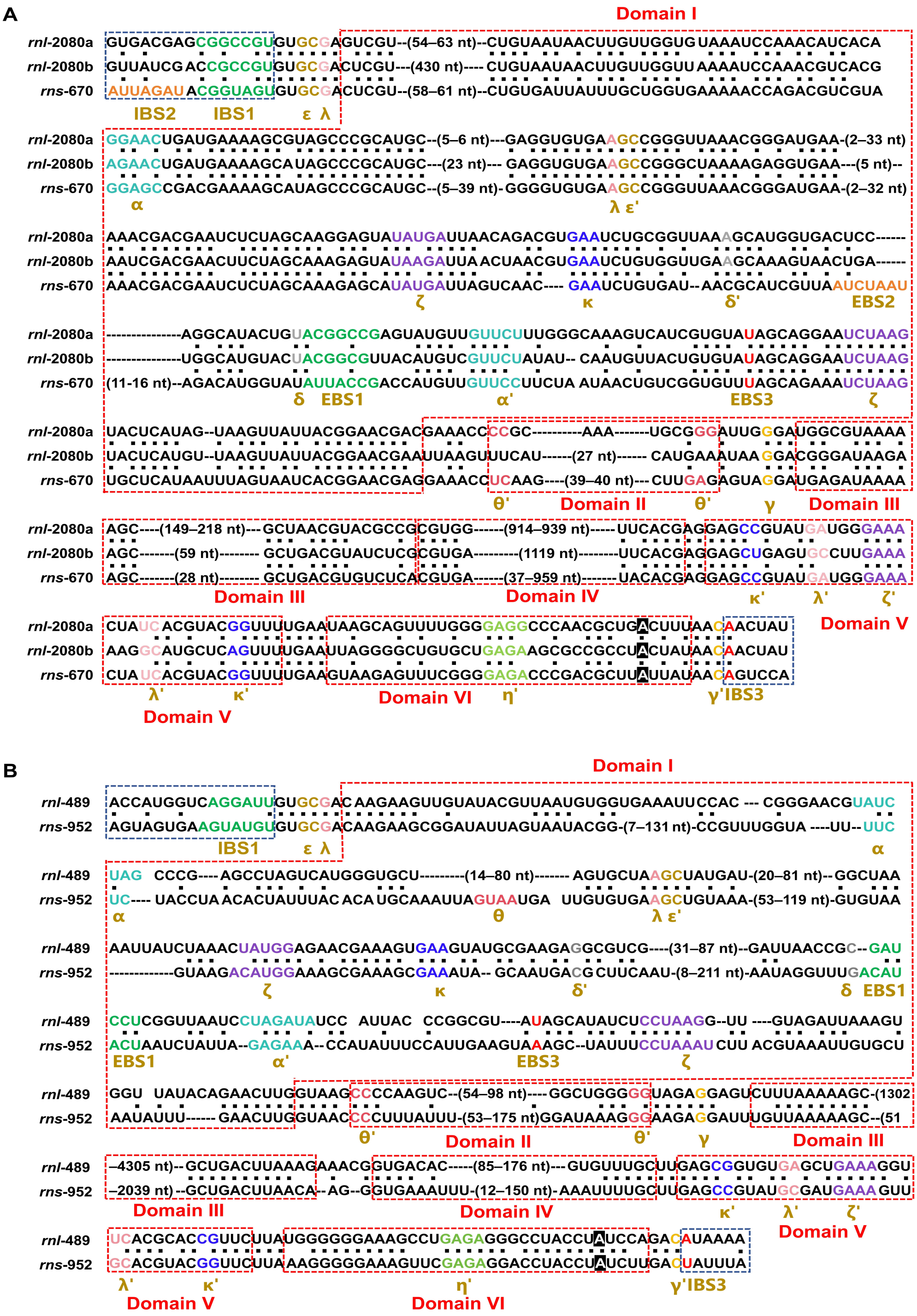

Supplementary Fig. S5 Secondary structure–guided sequence alignment of mitochondrial group II-LHE introns from green algae and fungi. (A) Group IIB2-LHE introns from green algae: *rnl*-2080a (found in *Ulva* species), *rnl*-2080b (found in *Gloeotilopsis* species) and *rns*-670. (B) Group IIB1-LHE introns from fungi: *rnl*-489 and *rns*-952. Exon sequences at the 5′ and 3′ termini are shown in uppercase letters. Intron sequences were aligned manually according to conserved secondary

structure domains. Canonical structural domains of group II introns (DI–DVI) are indicated with Roman numerals. Conserved tertiary interaction motifs, including IBS/EBS pairs (IBS1/EBS1, IBS2/EBS2, and IBS3/EBS3) and additional paired motifs (α–α′, β–β′, γ–γ′, κ–κ′, λ–λ′, δ–δ′, and ε–ε′), are labeled if present. The 5′ terminal linker is also indicated. Dashes represent alignment gaps introduced to maximize positional homology. Numbers in parentheses indicate nucleotide length variation (nt) among introns. Conserved nucleotides are color-coded according to their functional motif.

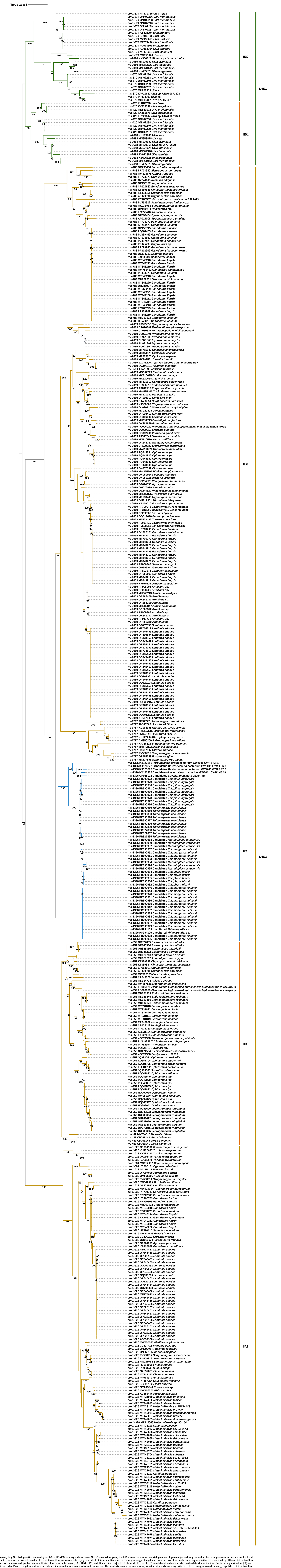

Supplementary Fig. S6 Phylogenetic relationships of LAGLIDADG homing endonucleases (LHE) encoded by group II-LHE introns from mitochondrial genomes of green algae and fungi as well as bacterial genomes. A maximum-likelihood phylogenetic tree was constructed based on LHE amino-acid sequences encoded by group II-LHE intron families across diverse green algal, fungal, and bacterial taxa. The tree includes representative LHE encoded by different intron families with accession numbers and species names indicated. The intron subclasses (IIA1, IIB1, IIB2, and IIC) and two major LHE clades (LHE1 and LHE2) are labeled and color-coded on the right side of the tree. Bootstrap support values (%) are shown at the nodes. Branch lengths are drawn to scale and the scale bar represents substitutions per site. This analysis reveals the evolutionary relationships and clustering patterns of LHE lineages from different group II-LHE intron families across different genomic contexts and taxonomic lineages.
